# A High‐Performance and Fully Recyclable Supramolecular Nanofibrous Membrane for Multifunctional Air Filtration

**DOI:** 10.1002/advs.202523204

**Published:** 2026-03-18

**Authors:** Wenjing Sun, Ding Yuan, Jiaqi Wan, Junyu Chen, Zhen Yan, Yinuo Yan, Senjie Dong

**Affiliations:** ^1^ Industrial Research Institute of Nonwovens and Technical Textiles Shandong Engineering Research Center for Specialty Nonwoven Materials College of Textiles and Clothing Qingdao University Qingdao P. R. China

**Keywords:** air filtration, closed‐loop recycling, multifunctional materials, nanofibrous membranes, supramolecular self‐assembly

## Abstract

Developing recyclable, multifunctional air purification materials that minimize environmental impact represents a grand challenge in the pursuit of carbon neutrality. Herein, a multifunctional air filtration membrane is prepared through a thermally induced precursor crystallization (TIPC) process that drives the self‐assembly of a supramolecular nanofibrous network from melamine (MA) and trimesic acid (TMA). Graphene oxide (GO) nanosheets, strategically incorporated as heterogeneous nucleation‐templating agents, are critical for tailoring the hierarchical network morphology and endowing the membrane with multiple synergistic properties. The resulting membrane delivers exceptional particulate matter (PM) filtration efficiency (99.48% for PM_1.0_), potent antimicrobial activity against (*Escherichia coli*) *E. coli* (99.4%) and (*Staphylococcus aureus*) *S. aureus* (99.8%), and a high formaldehyde removal capacity (95%). Beyond purification, the membrane also demonstrates effective thermal management for wearer comfort and superior UV‐blocking (< 0.4% transmittance). Crucially, the inherent thermoreversibility of the supramolecular network enables complete, closed‐loop recycling of the membrane using only green solvents under mild conditions. This work presents a comprehensive and sustainable strategy for preparing advanced air filtration materials, demonstrating that high performance, integrated multifunctionality, and complete lifecycle circularity can be co‐designed from the molecular level up.

## Graphical abstract

A green, thermoreversible self‐assembly strategy yields a multifunctional and recyclable supramolecular nanofibrous membrane. Graphene oxide (GO) nanosheet acts as a heterogeneous nucleation‐templating agent, simultaneously modulating the supramolecular crystal packing while tailoring the nanofibrous architecture for elite performance. The resulting membrane provides exceptional PM filtration, antimicrobial activity, HCHO capture, robust UV‐shielding, and effective thermal management, all while retaining complete closed‐loop recyclability.

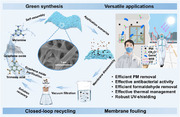

## 1 Introduction

Air pollution constitutes a severe global health crisis, responsible for an estimated 4–9 million premature deaths annually [1, 2]. The threat is exacerbated by the complex nature of contemporary airborne contaminants—a synergistic mixture of particulate matter (PM), pathogenic microorganisms (e.g., bacteria, viruses), and volatile organic compounds (VOCs) [3, 4]. This multifaceted health challenge has catalyzed intensive research into the development of advanced filtration materials [5, 6]. However, the rational design of multifunctional filters capable of simultaneously and efficiently neutralizing such a diverse range of pollutants remains a formidable scientific challenge. Furthermore, it is imperative to look beyond filtration performance and address the environmental impact of the materials throughout their entire lifecycle, necessitating a shift toward truly sustainable solutions.

Fiber‐based materials, prized for their high specific surface area, tunable porosity, and structural versatility, represent the cornerstone of modern air purification technologies [7, 8, 9]. Their widespread application, however, is predicated on conventional manufacturing paradigms—such as melt‐blowing, electrospinning, and spun‐lacing—that are fraught with inherent drawbacks, including high energy consumption and the extensive use of hazardous solvents [10, 11]. The predominant reliance on petroleum‐based polymers further exacerbates this challenge; their fossil origin and non‐biodegradable nature lead to persistent microplastic pollution and long‐term ecological risks upon disposal [12, 13]. While bio‐based polymers like polylactic acid (PLA) are often presented as a solution [14], they face significant scalability hurdles; their high production cost renders direct degradation economically irrational, while slow natural decomposition rates and inefficient recycling pathways limit their practical circularity [15, 16, 17]. A further layer of complexity arises from the growing trend of incorporating nanomaterials to impart multifunctionality (e.g., antimicrobial, adsorption, or catalytic properties) [18]. The end‐of‐life disposal of these nanocomposite filters presents a dual dilemma: (i) the irreversible loss of high‐value functional nanomaterials, and (ii) the potential leakage of these nanomaterials into ecosystems, posing unquantified yet significant risks to environmental and human health [19, 20]. This cascade of performance limitations and sustainability challenges underscores an urgent and unmet need: to transition towards a new generation of recyclable, high‐performance filtration materials engineered through fundamentally green manufacturing processes and designed for complete circularity.

Supramolecular self‐assembly represents a molecular self‐organization process driven by noncovalent interactions, wherein building blocks spontaneously organize into structurally well‐defined organic crystalline materials through directional forces such as hydrogen bonding, van der Waals forces, and π–π stacking [21, 22, 23]. This bottom‐up design strategy exhibits several distinctive advantages: (i) precise hierarchical control spanning molecular to macroscopic scale; (ii) dynamic reversibility of intermolecular interactions enabling material reconfigurability and recycling; (iii) broad adaptability for diverse applications [24, 25, 26, 27]. These unique characteristics have established this approach as a prominent research frontier in filtration materials science. Schmidt et al. [28, 29] successfully constructed nonwoven filtration materials with hierarchical mesostructures by integrating 1,3,5‐benzenetricarboxamides self‐assembled nanofibers with electrospun fibers. Zhuang et al. [30, 31] respectively combined supramolecular self‐assembly with solution blowing and melt‐blown technologies to fabricate a hierarchical dual‐nanofiber network, achieving high PM filtration efficiency and low pressure drop. However, these hybrid approaches still rely on the conventional polymer matrix for structural integrity and, critically, fail to leverage the intrinsic dynamic reversibility of supramolecular systems for material recycling, thus only partially addressing the sustainability challenge.

To bridge this gap, our group previously developed the thermally induced precursor crystallization (TIPC) strategy, enabling the preparation of freestanding and fully recyclable supramolecular nanofiber membranes assembled from melamine (MA) and trimesic acid (TMA) [32]. While the incorporation of functional nanomaterials like silica nanoparticles (SiO_2_ NPs) and covalent organic frameworks (COFs) was achieved to create high‐performance air filters [33, 34], these initial explorations are limited in two key aspects: (i) a deep mechanistic understanding of the nanoparticle‐nanofiber interactions governing the final microstructure is lacking, and (ii) the resulting supramolecular composites exhibit relatively unidimensional functionality. By elucidating the self‐assembly mechanisms of supramolecular composites, it is promising to construct air purification materials that integrate complete recyclability with synergistic multifunctionality, thereby opening new pathways for bottom‐up design of sustainable materials.

**FIGURE 1:**
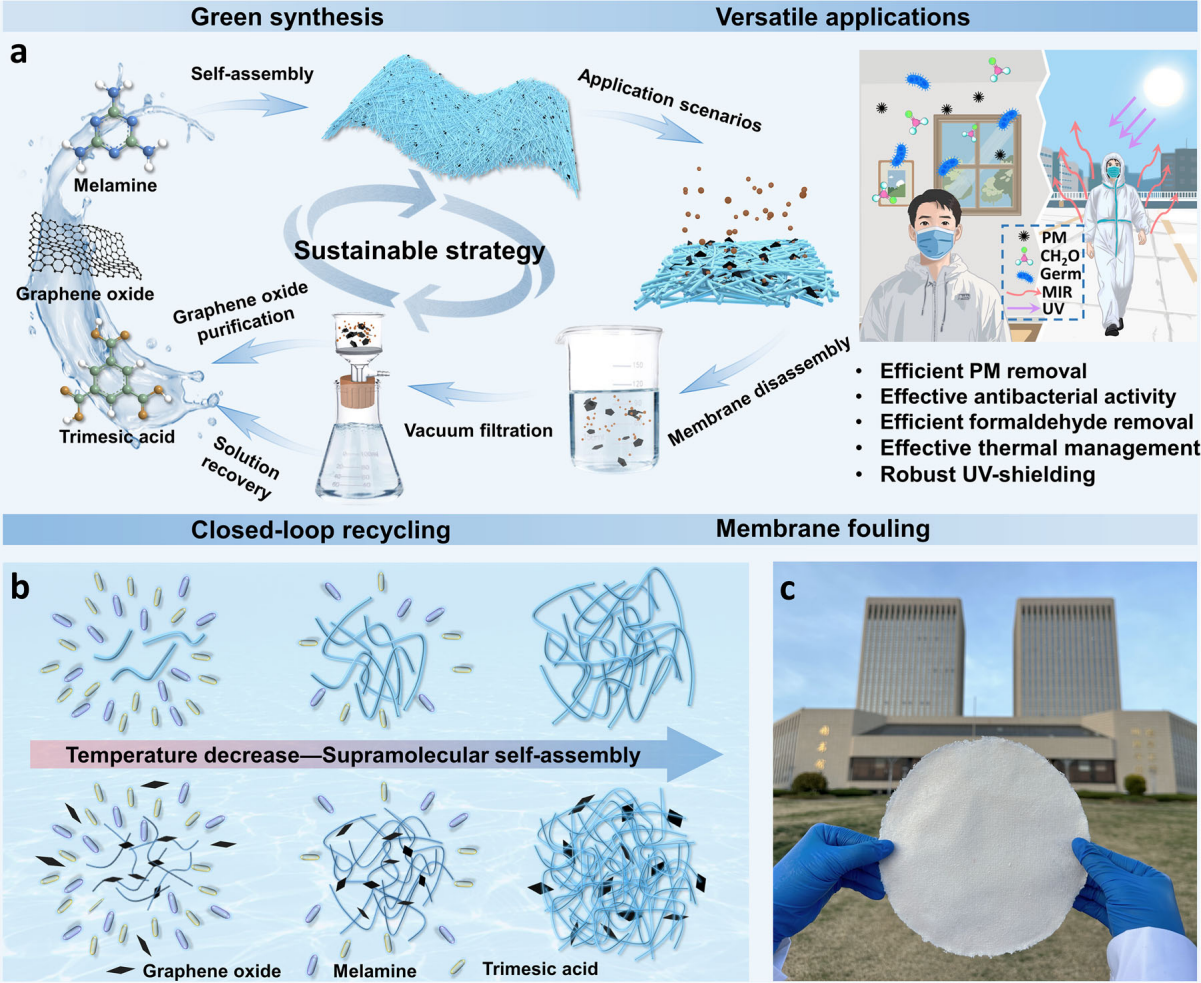
Design concept, self‐assembly mechanism, and physical appearance of the GO@MA·TMA membrane. (a) Schematic illustration of the preparation process, multifunctional application scenarios, and closed‐loop recycling strategy of the GO@MA·TMA membrane. (b) Schematic depicting the temperature‐dependent self‐assembly of the 3D supramolecular nanofibrous network of both MA·TMA and GO@MA·TMA. (c) Photograph of a large‐area, freestanding GO@MA·TMA membrane.

Herein, we report the preparation of a multifunctional and recyclable graphene oxide‐integrated supramolecular nanofiber (GO@MA·TMA) membrane for sustainable filtration (Figure 1). By introducing GO nanosheets as heterogeneous nucleation‐templating agents [35, 36], we harness their abundant surface functional groups (e.g., hydroxyl, carboxyl) and π‐conjugated domains to precisely control the self‐assembly of the MA·TMA supramolecular system via robust hydrogen bonding and π–π stacking interactions. This rational design strategy allows for the engineering of a hierarchical nanofibrous architecture that endows the membrane with a remarkable suite of functionalities, including ultrahigh filtration efficiency, broad‐spectrum antimicrobial activity, efficient formaldehyde (HCHO) capture, effective thermal management, and robust UV‐shielding. Notably, the inherent thermoreversibility of the supramolecular network enables the membrane to be completely dissolved and reconstructed, establishing a closed‐loop recycling pathway that ensures component recovery. This work presents a paradigm for designing next‐generation sustainable air purification materials where elite performance and complete lifecycle circularity are realized simultaneously.

## 2 Results and Discussion

Figure 1a schematically illustrates the complete lifecycle of the GO@MA·TMA membrane, representing a holistic sustainable strategy that seamlessly integrates its green synthesis from fundamental molecular building blocks (MA and TMA) and functional GO nanosheets, through its versatile applications in diverse air purification scenarios, to its ultimate closed‐loop recycling via membrane disassembly and component recovery. This comprehensive design minimizes environmental impact across the entire material's lifespan.

Fundamentally, the underlying mechanism for the green synthesis, detailed in Figure S1, is a two‐stage thermally induced precursor crystallization (TIPC) process driven by cooperative hydrogen bonding and *π*–*π* stacking interactions between MA and TMA monomers [32]. In the absence of a dedicated template (Figure 1b, top panel), this process relies on spontaneous homogeneous nucleation, often leading to poor structural uniformity. While any foreign substrates (such as particulate contaminants, bubbles, or pre‐existing nanocrystalline fibers) can induce heterogeneous nucleation [24] in the MA·TMA supramolecular system to initiate nanofiber growth, the key innovation in this work (Figure 1b, bottom panel) lies in the incorporation of GO nanosheets. By serving as heterogeneous nucleation‐templating agents, the uniformly dispersed GO nanosheets reduce the nucleation energy barrier for the self‐assembling monomers through an adsorption‐mediated mechanism, thereby increasing the nucleation density [35, 37, 38, 39]. This strategic intervention effectively suppresses radial fiber growth, enabling precise control over the fiber diameter of GO@MA·TMA membrane. This proposed mechanism is subsequently corroborated by scanning electron microscopy (SEM) and validated at the atomic level by density functional theory (DFT) calculations.

This bottom‐up self‐assembly process drives the anisotropic growth of precursors into ultralong, nanocrystalline fibers with high‐aspect‐ratios, which form a freestanding membrane upon drying (Figure 1c). The GO@MA·TMA membrane exhibited a tensile strength of 1.6 ± 0.2 MPa and an elongation at break of 2.3 ± 0.3%, while retaining excellent flexibility for practical handling (Figure S2). Notably, the entire synthesis is conducted under mild conditions using only green solvents (ethanol and water), bypassing the need for sophisticated equipment.

A key attribute of the GO@MA·TMA system, stemming from the dynamic nature of its supramolecular interactions, is its inherent thermoreversibility. This equilibrium‐driven behavior is characterized by a fully reversible structural transition: the nanocrystalline fibers spontaneously assemble upon cooling and completely dissolve back into precursors upon heating (Figure S3). This property underpins the GO@MA·TMA membrane's complete recyclability. Leveraging the equilibrium‐reversible nature of the GO@MA·TMA system, a three‐stage, closed‐loop recycling protocol is established (Figure S4): (i) Thermal dissolution and purification, where the used composite membrane is dissolved by heating and solid contaminants are removed via filtration; (ii) Component recovery, involving the separation and purification of the GO nanosheets and the MA·TMA precursor solution; and (iii) Membrane reconstitution, where the recovered components are recombined and subjected to the TIPC process to form a new composite membrane. In the recycling process, the MA·TMA nanocrystalline fibrous network is readily re‐dissolved to reconstitute a saturated precursor solution. This “solution‐to‐solution” closed‐loop strategy facilitates a recovery yield of ∼99% (virtually lossless), permitting the direct re‐deployment of the MA·TMA supramolecular complex in subsequent TIPC cycles. Meanwhile, the isolation of GO from trapped PM is achieved via an ultrasonic‐centrifugal purification protocol. This process exploits the distinct disparity between the intrinsic colloidal stability of GO nanosheets and the rapid sedimentation behavior of aggregated PM contaminants. The sonication‐assisted treatment effectively disrupts interfacial adhesion, yielding recovered GO with a quantitative yield of ∼91%. The marginal mass loss is primarily attributed to the mechanical loss during the laboratory scale treatment, i.e., filtration, washing, drying, and transfer. SEM and FTIR spectroscopy analyses confirmed that the recycled membrane was virtually indistinguishable from its pristine counterpart, retaining its original nanofibrous architecture and chemical structure (Figures S5 and S6).

Crucially, the molecular‐level self‐assembly of MA·TMA, strategically directed by the introduction of GO nanosheets as heterogeneous nucleation templates, not only confers the GO@MA·TMA supramolecular system's inherent thermoreversibility and full recyclability, but also enables the engineering of a tailored composite micro‐architecture that intrinsically yields exceptional multifunctional performance. As a filtration medium, the GO@MA·TMA membrane demonstrates exceptional PM capture efficiency, removing 99.48% of PM_1.0_, 99.96% of PM_2.5_, and 99.98% of PM_10_. Beyond filtration, the synergistic integration of GO nanosheets and the MA·TMA supramolecular matrix imparts a suite of advanced functions: (i) broad‐spectrum antimicrobial activity (99.4% inhibition against *E. coli* and 99.8% against *S. aureus*); (ii) enhanced toxic gas adsorption (95% HCHO removal); (iii) UV‐shielding capability (< 0.4% transmittance at 280–400 nm); and (iv) effective thermal management (maintaining a surface temperature of 32°C close to human body temperature). This work establishes a sustainable materials paradigm through an eco‐friendly, low‐energy process, integrating multifunctional performance with full life‐cycle recyclability from design and fabrication to closed‐loop regeneration, thereby providing a blueprint for next‐generation air filtration materials.

**FIGURE 2:**
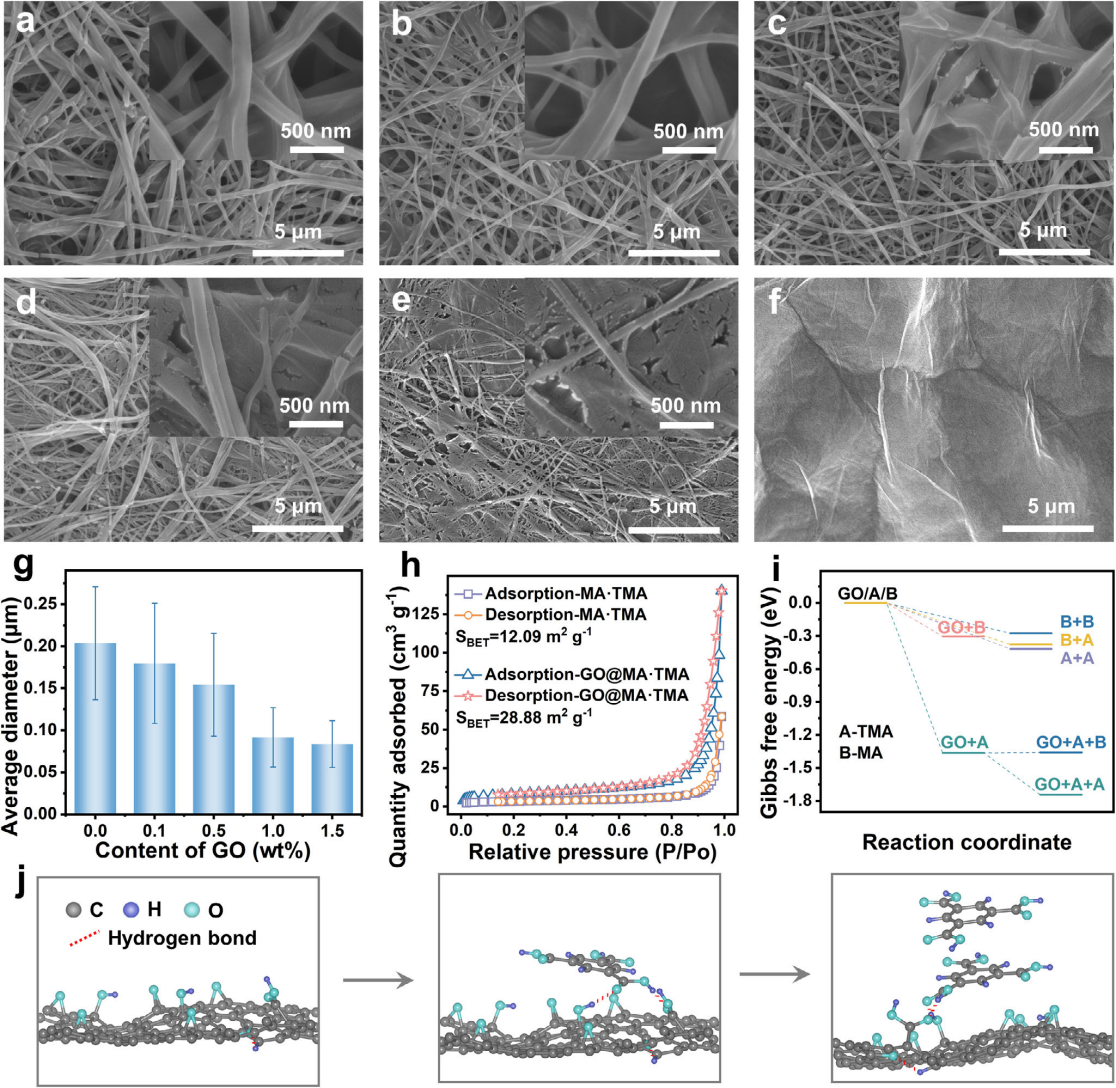
Microstructural characterization and mechanistic investigation of the GO‐modulated supramolecular self‐assembly. (a–e) SEM images showing the morphological evolution of GO@MA·TMA membranes with increasing GO concentration: (a) 0 wt.%, (b) 0.1 wt.%, (c) 0.5 wt.%, (d) 1.0 wt.%, and (e) 1.5 wt.%. (f) SEM image of GO nanosheets. (g) Corresponding plot of the average fiber diameter as a function of GO concentration. (h) Comparative nitrogen adsorption‐desorption isotherms for pristine MA·TMA membrane and GO@MA·TMA membrane. (i) DFT‐calculated energy profiles for the nucleation of MA·TMA supramolecular precursors with and without the presence of a GO nanosheet. (j) Schematic of the proposed energy‐favorable nucleation pathway on the GO surface.

Given the critical role of microstructure in determining the performance attributes of GO@MA·TMA membranes, a comprehensive microstructural characterization was conducted using SEM to elucidate the morphological evolution of membranes (Figure 2a–e). The pristine MA·TMA membrane consisted of randomly oriented, smooth‐surfaced nanocrystalline fibers (Figure 2a), consistent with prior observations of self‐assembly branching phenomena [24, 32]. In contrast, the GO nanosheets (Figure 2f), possessing a characteristic 2D geometry and high specific surface area essential for PM capture [40], induced a significant microstructure transformation of the membrane. Upon incorporation, the composite membranes exhibited increased surface roughness, forming an interpenetrating network of MA·TMA nanocrystalline fibers and GO nanosheets (Figure 2b–e). Here, the MA·TMA nanocrystalline fibers act as a structural scaffold, while the GO nanosheets form bridge structure between adjacent fibers. This bridging structure modulates pore connectivity and size distribution, thereby enhancing the capture capacity for pollutants.

Notably, increasing the GO content led to a more uniform fiber diameter distribution and a progressive reduction in average fiber diameter (Figure S7). Specifically, the average fiber diameter of the composite membrane containing 0.5 wt.% GO is reduced by 25% (from 204 to 154 nm) compared to the pristine membrane (Figure 2g). The microstructure evolution of the GO@MA·TMA membranes was further investigated by N_2_ sorption at 77 K. Brunauer–Emmett–Teller (BET) analysis confirmed both membranes were mesoporous (type IV isotherms), but GO incorporation increased the specific surface area (*S*_BET_) by a remarkable 139% (from 12.09 to 28.88 m^2^ g^−1^) (Figure 2h). This corresponds to a substantial increase in the proportion of small mesopores (< 20 nm) (Figure S8), a critical factor for enhancing PM removal efficiency.

To elucidate the mechanism of this GO‐induced fiber diameter regulation, DFT calculations were performed to analyze the Gibbs free energy (ΔG) of the initial nucleation stage. The calculations revealed that GO nanosheets provided highly favorable adsorption sites for the MA·TMA supramolecular precursors. As shown in Figure 2i, the ΔG for MA and TMA adsorption on the GO surface was −0.30 and −1.36 eV, respectively. Notably, the energy change for TMA on the GO surface (ΔG = −1.36 eV) was significantly more negative than that of the intermolecular interactions in the pristine system (MA‐MA: −0.28 eV; MA‐TMA: −0.42 eV; TMA‐TMA: −0.38 eV). This pronounced negative energy shift confirmed that GO nanosheets drastically lowered the nucleation barrier via enhanced interfacial interactions [35]. Further simulations (Figure 2j; Figure S9) showed that subsequent TMA adsorption onto a pre‐adsorbed GO‐TMA complex (ΔG = −0.39 eV) was primarily driven by π–π stacking, which is also a key driving force for the self‐assembly of MA‐TMA supramolecular system [32, 41]. From the results of Gibbs energy changes, it can be concluded that the self‐assembly of MA‐TMA nanocrystalline fibers is a spontaneous process and thermodynamically more favorable on the GO surface (Note S1). Based on these observations, we suggest that GO nanosheets facilitate heterogeneous nucleation, thereby increasing nucleation density [37] and restricting radial fiber growth, which ultimately results in finer fiber diameters (Note S2). This mechanism is consistent with the observed inverse correlation between GO concentration and average fiber diameter in SEM images. This strategy demonstrates that 2D nanosheets can be employed for the tailored modulation of 3D supramolecular nanofibrous networks, providing a novel pathway for designing advanced functional materials.

**FIGURE 3:**
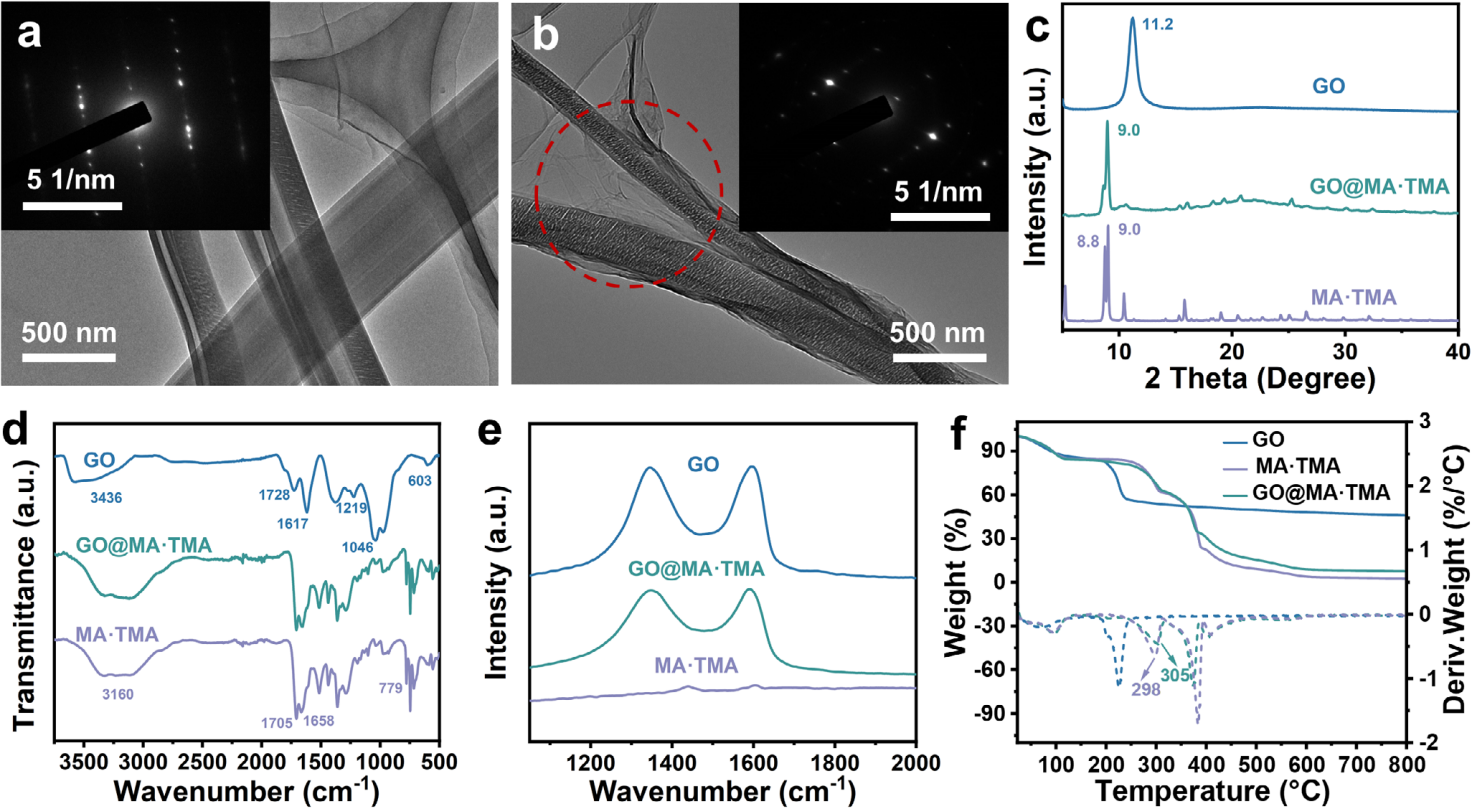
Comprehensive microstructural, chemical, and thermal characterization of membranes. (a,b) TEM images of pristine MA·TMA membrane and GO@MA·TMA membrane; insets show their corresponding SAED patterns. (c–f) Comparative analyses of GO nanosheet, MA·TMA membrane, and GO@MA·TMA membrane via: XRD patterns, FTIR spectra, Raman spectra, and TGA and DTG curves.

Transmission electron microscopy (TEM) further elucidated the intimate interfacial morphology between GO nanosheets and MA·TMA nanocrystalline fibers. While the pristine MA·TMA nanofibers possessed smooth surfaces (Figure 3a), the GO@MA·TMA composite was uniformly coated with monolayer GO nanosheets, which introduced surface wrinkles and bridged neighboring fibers (Figure 3b, red circles). These observations corroborate the SEM findings, confirming the homogeneous dispersion and strong interfacial integration of GO nanosheets within MA·TMA supramolecular nanofibers. Simultaneously, the appearance of clear lattice fringes also revealed that both nanofibers had a crystalline structure. The corresponding selected‐area electron diffraction (SAED) patterns (Figure 3a,b, insets) showed d‐spacings of 3.46 Å (pristine fibers) and 3.45 Å (composite fibers) along the fiber axis, which matched the π–π stacking distance of MA·TMA supramolecular complexes [32, 41]. This observation indicates a highly oriented molecular arrangement where the π–π stacking direction is aligned parallel to the nanocrystalline fibers axis.

X‐ray diffraction (XRD) analysis also provided definitive evidence for the successful integration of GO nanosheets into MA·TMA supramolecular complexes. As clearly demonstrated in Figure 3c, the pristine MA·TMA membrane exhibited two sharp diffraction peaks at 8.8° and 9.0°, indicative of a highly crystalline, well‐ordered supramolecular structure [32, 33, 34]. In parallel, the GO nanosheets exhibited their characteristic peak at 11.2°, corresponding to an expanded interlayer spacing of 8 Å according to Bragg's law, indicating the successful introduction of abundant oxygen‐containing functional groups (e.g., hydroxyl, epoxy, and carboxyl groups) between the graphene layers [42]. These oxygen‐containing functional groups not only enable GO nanosheets to serve as heterogeneous nucleation‐templating agents but also play a critical role in pollutant adsorption through multiple mechanisms [43]. Notably, the incorporation of GO nanosheets induced significant changes in the composite membrane's XRD pattern, manifested as a merging trend of the two diffraction peaks at 8.8° and 9.0°, along with a reduction in the intensity of the peak at 8.8°. These observations clearly indicated reduced crystallinity and reduced crystallite size in the GO@MA·TMA membrane compared to the pristine MA·TMA membrane. This structural modification suggests that GO nanosheets can effectively modulate the crystalline self‐assembly of the MA·TMA supramolecular complex.

Fourier transform infrared spectroscopy (FTIR) was employed to probe the chemical structures of GO nanosheet, MA·TMA membrane, and GO@MA·TMA membrane (Figure 3d). The spectrum of GO nanosheet displayed characteristic absorption bands for O–H stretching (broad, 3436 cm^−^^1^), C═O stretching (1728 cm^−^^1^), C═C vibrations (1617 cm^−^^1^) and various C–O stretching vibrations from carboxyl, epoxy, and alcoholic groups [44], confirming the abundance of oxygen−containing functional groups previously inferred from XRD data. For the pristine MA·TMA membrane, the broad absorption band at 3160 cm^−^^1^ corresponds to O─H stretching vibrations of water molecules embedded within the hydrogen‐bonded network. The characteristic vibrational signatures at 1705 cm^−^^1^ (νC═O), 1658 cm^−^^1^ (νC═C), and 779 cm^−^^1^ (σtriazine) provide definitive evidence for the MA·TMA supramolecular complex formation [32, 33, 34]. Comparative vibrational spectroscopic analysis demonstrates that the intrinsic molecular signatures of MA·TMA remain preserved in the GO@MA·TMA membrane, confirming that the introduction of a small amount of GO nanosheets does not significantly disrupt the framework of MA·TMA supramolecular complex. Raman spectroscopy further provided definitive proof of GO nanosheet's successful incorporation. The GO@MA·TMA membrane spectrum (Figure 3e) exhibited two characteristic strong peaks: the D‐band at approximately 1350 cm^−^^1^ and the G‐band at approximately 1590 cm^−^^1^. The D‐band is typically attributed to the breathing modes of sp^2^ rings, which become Raman active due to edges or structural defects in the graphene oxide lattice; while the G‐band corresponds to the first‐order scattering of sp^2^ hybridized carbon domains within the GO structure [45, 46].

Thermal stability is a critical parameter for air filters in practical applications. Figure 3f presents the thermogravimetric (TG, solid lines) and derivative thermogravimetric (DTG, dashed lines) curves of GO nanosheet, MA·TMA membrane, and GO@MA·TMA membrane. Below 100°C, all materials showed a minor mass loss attributed to the removal of adsorbed molecular water. GO nanosheet exhibited a distinct decomposition event at 227°C (Δm = 26.3%), assigned to the pyrolysis of its abundant oxygen‐containing functional groups [46, 47]. The pristine MA·TMA membrane, in turn, demonstrated a multi‐stage mass loss profile: initial water evaporation (∼94°C), followed by two primary decomposition stages at 298°C and 383°C, attributed to the thermal degradation of the MA and TMA molecular components, respectively [32, 33, 34]. Significantly, the GO@MA·TMA membrane demonstrated enhanced thermal stability. While its degradation profile was similar to the pristine membrane, DTG analysis revealed the primary decomposition peak was shifted to a higher temperature of 305°C (versus 298°C for the pristine membrane). This positive shift clearly indicates that the well‐integrated GO nanosheets reinforce the structural integrity of the MA·TMA supramolecular framework. The GO@MA·TMA membrane thus exhibits excellent thermal robustness for applications operating below the initial pyrolysis temperature of GO nanosheets (∼227°C).

**FIGURE 4:**
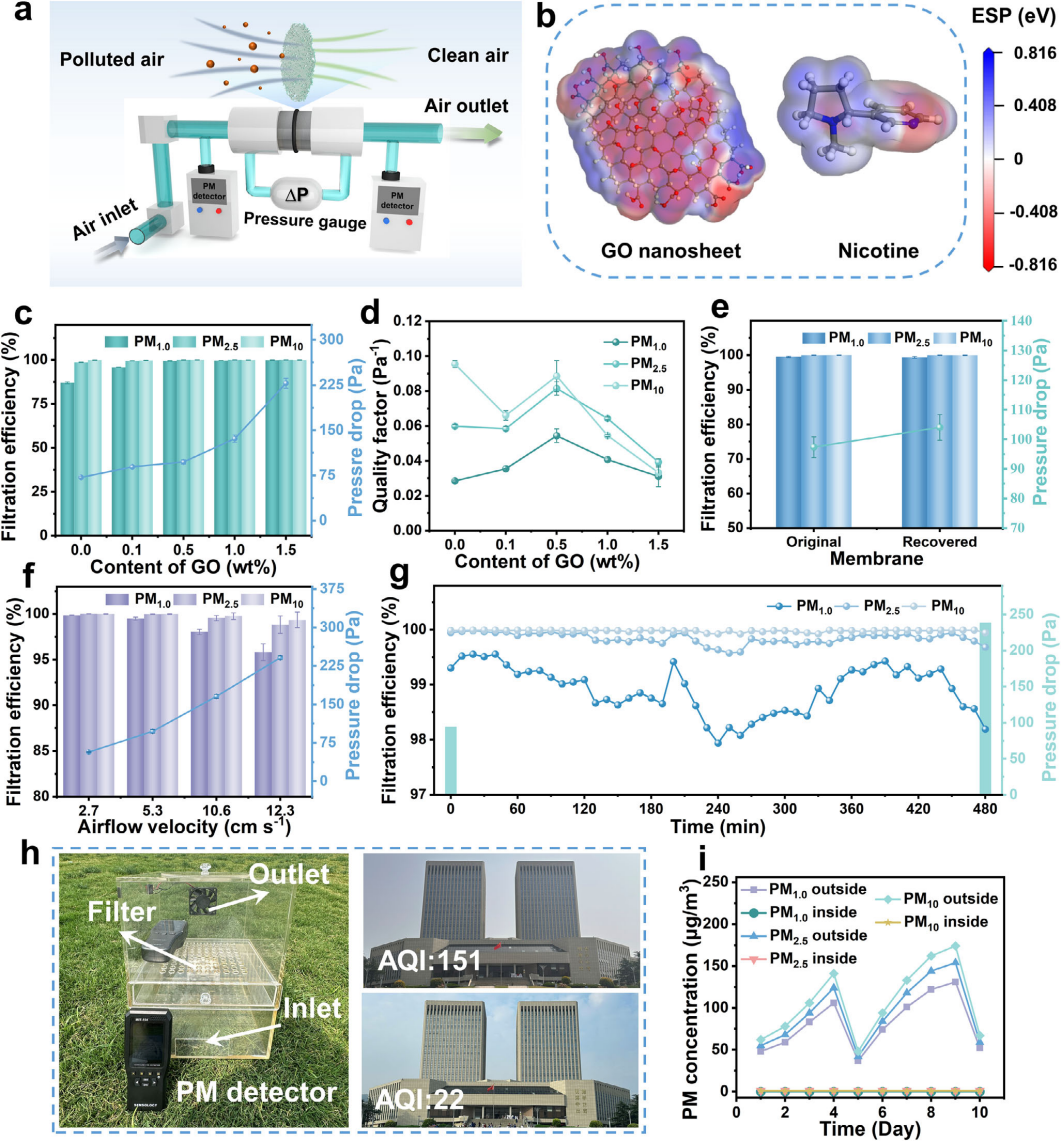
Comprehensive air filtration performance, mechanistic insight, and practical application of the GO@MA·TMA membrane. (a) Schematic diagram of the air filtration test setup. (b) ESP mapping of a GO nanosheet and a nicotine molecule. (c) Filtration performance and (d) corresponding QF value for GO@MA·TMA membranes as a function of GO concentration. (e) Comparative filtration performance of the origin and recycled GO@MA·TMA membranes. (f) Filtration performance of 0.5 wt.% GO@MA·TMA membranes under various airflow velocities. (g) Long‐term filtration stability of the 0.5 wt.% GO@MA·TMA membrane over an 8‐h continuous test. (h,i) Photographs of the practical filtration device and test environment, and the corresponding 10‐day field test performance.

To assess the GO@MA TMA membrane's versatility against complex air pollutants, cigarette smoke—which is compositionally similar to haze pollution and challenging to capture—was used as the aerosol source. As shown in Figure 4a, polluted air passes through an initial detector, the filter membrane, and a second detector, while the pressure drop is monitored in real‐time. Electrostatic surface potential (ESP) calculations (Figure 4b) confirmed that the abundant oxygen‐containing functional groups on the GO nanosheets created a strong negative potential surface. This results in strong electrostatic attraction toward the positive potential regions of polar molecules like nicotine, providing the mechanistic basis for the membrane's enhanced pollutant capture. The composite membrane's performance was optimized by varying the GO loading. As shown in Figure 4c, the filtration performance of x wt.% GO@MA·TMA membranes were evaluated at a fixed basis weight (10 g m^−2^) and airflow velocity (5.3 cm s^−1^). The pristine MA·TMA membrane exhibited a pressure drop of 72 Pa with filtration efficiencies of 86.97%, 98.63%, and 99.89% for PM_1.0_, PM_2.5_, and PM_10_, respectively. In striking contrast, the 0.5 wt.% GO@MA·TMA membrane demonstrated exceptional performance, achieving removal efficiencies of 99.48%, 99.96%, and 99.98% for PM_1.0_, PM_2.5_, and PM_10_, respectively, with only a modest increase in pressure drop to 97 Pa, which is substantially below the NIOSH (National Institute for Occupational Safety and Health) regulatory limit (∼320 Pa). With increasing GO loading, the GO@MA·TMA membranes exhibited diminishing returns in filtration efficiency enhancement, accompanied by a substantial rise in pressure drop, which is likely attributed to excessive coverage and blockage of inter‐fiber pores by GO nanosheets.

To provide a comprehensive metric of performance, the quality factor (QF) was calculated for each membrane. As illustrated in Figure 4d, the 0.5 wt.% GO@MA·TMA membrane demonstrated optimal filtration performance, with QF of 0.054, 0.082, and 0.089 Pa^−^^1^ for PM_1.0_, PM_2.5_, and PM_10_, respectively. These impressive QF values are not only comparable to those of many state‐of‐the‐art filters (Table S1), but are also complemented by the material's unparalleled recyclability. This complete recyclability is directly enabled by the thermoreversible nature of the MA·TMA supramolecular system (Figure S3). Notably, after a full dissolution and reconstitution cycle (Figure S4), the recycled 0.5 wt.% GO@MA·TMA membrane demonstrated nearly identical performance to its original counterpart, achieving filtration efficiencies of 99.32% (PM_1.0_), 99.96% (PM_2.5_), and 99.95% (PM_10_) with only a negligible increase in pressure drop of 7 Pa (Figure 4e). Overall, the recycled membrane retained > 99.84% of its original removal efficiency for all particle sizes. Unlike traditional recyclable filters that suffer from performance fatigue due to the accumulation of irreversible mechanical damage during physical washing cycles (Table S2), the GO@MA‐TMA membrane leverages a “solution‐to‐solution” reconstruction strategy. This process harnesses the reversible nature of supramolecular interactions to erase structural defects and contamination history, ensuring that the regenerated membrane theoretically maintains the pristine structural integrity and filtration performance of a newly fabricated one, regardless of the cycle count.

The exceptional filtration performance of the GO@MA·TMA membrane originates from a synergy between enhanced passive mechanical filtration and active electrostatic adsorption. Classical filtration theory dictates that passive capture—primarily comprising physical interception, inertial impaction, and Brownian diffusion—is critically dependent on factors like fiber diameter and particle size [8, 18]. As confirmed by our microstructural analysis, the incorporation of GO nanosheets induces the heterogeneous nucleation of MA·TMA supramolecular system, leading to a reduced average fiber diameter and a significantly higher specific surface area. The resulting bridging structure formed by GO nanosheets between MA·TMA nanocrystalline fibers directly enhances the probability of particle capture via both interception and diffusion mechanisms. In addition, electrostatic attraction represents a powerful active capture mechanism. The GO@MA·TMA membrane is engineered to maximize this effect through a dual‐mode chemical functionality. The abundant, high‐dipole oxygen‐containing functional groups on the integrated GO nanosheets serve two distinct roles: [40, 43] (i) they create a strong negative potential surface, as confirmed by ESP calculations (Figure 4b), which generates potent electrostatic attraction toward the positive potential regions of polar PM, and (ii) they carry a net negative charge (e.g., deprotonated carboxyls) that generates a more potent, long‐range electrostatic field. This potent electrostatic effect was visibly demonstrated at the macro‐scale, where a 0.5 wt.% GO@MA·TMA membrane readily adsorbed materials like insulating carbon powder and feathers via strong static attraction (Figure S10). Thus, the GO@MA·TMA membrane synergistically combines an optimized physical structure for mechanical capture with active chemical functionality for potent electrostatic adsorption (Figure S11).

Environmental adaptability and long‐term stability are indispensable metrics for practical filtration materials. To this end, the 0.5 wt.% GO@MA·TMA membrane was subjected to extreme operational conditions. As illustrated in Figure 4f, the composite membrane maintained high‐efficiency filtration (> 95.81% for all PM sizes) across a wide range of airflow velocities (2.7 to 12.3 cm s^−^^1^), with the pressure drop reaching a modest 241 Pa at the maximum flow rate. Furthermore, the composite membrane demonstrated excellent resilience to moisture, retaining exceptional filtration performance (> 98.87% for all PM sizes) even under high relative humidity (RH) conditions (up to 90% RH) (Figure S12). Long‐term stability was then assessed via a continuous 8‐h test under an extreme aerosol load (PM_2.5_> 2000 µg m^−3^; PM_10_> 4000 µg m^−3^). Throughout the test, the composite membrane consistently maintained superior filtration efficiencies of > 99.57% for PM_2.5_/PM_10_ and > 97.93% for PM_1.0_ (Figure 4g). Concurrently, the pressure drops steadily increased from 97 to 238 Pa. This gradual rise confirms that the composite membrane effectively captures the high concentration of particles without structural degradation or failure, thus demonstrating its exceptional operational stability.

To validate its real‐world applicability, the 0.5 wt.% GO@MA·TMA membrane was subjected to a 10‐day outdoor field test under diverse ambient and meteorological conditions at Qingdao University (Figure 4h). As shown in Figure 4i, the composite membrane demonstrates robust filtration stability, consistently maintaining output PM concentrations at ultralow levels regardless of daily fluctuations in air quality or weather. This performance confirms the composite membrane's excellent reliability and suitability for practical, long‐term air purification applications.

Ideally, the structural optimization of supramolecular membranes has evolved from uncontrolled self‐assembly to precise template‐directed growth. While our initial pristine MA·TMA system established the TIPC protocol, it suffered from a high pressure drop (363 Pa) due to the lack of precise microstructural control [32]. Subsequent efforts using SiO_2_ NPs as 0D nucleation sites improved filtration efficiency (99.82% for PM_1.0_) but failed to resolve the resistance issue (153 Pa) caused by particle agglomeration [33]. Although TP‐BPY‐COF was later introduced to lower air resistance via its high surface area, their complex synthesis and high cost hinder economic scalability [34]. Distinct from previous 0D/2D hard templates, the oxygen‐rich and *π*‐conjugated GO nanosheets induce a refined crystallization of MA·TMA nanofibers, achieving an optimal balance between high efficiency (99.48% for PM_1.0_) and low pressure drop (97 Pa). The transition from “uncontrolled self‐assembly” to “0D/2D hard templates” and finally to the “2D flexible template” represents a logical scientific progression, establishing the GO@MA·TMA supramolecular system as a reliable solution for advanced air filtration.

**FIGURE 5:**
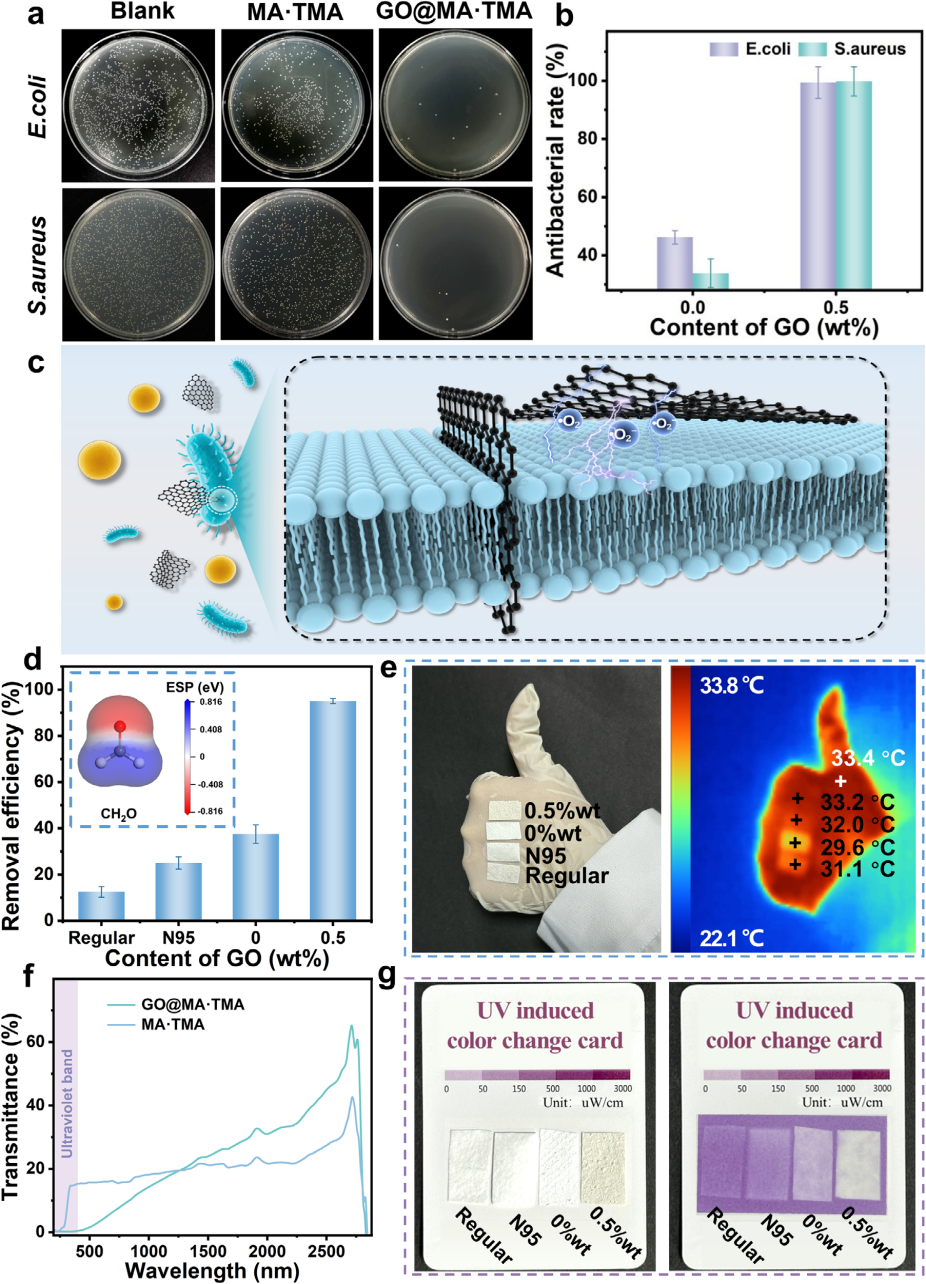
Multifunctional performance of the GO@MA·TMA membrane. (a) Photographs of bacterial colonies on Luria‐Bertani (LB) agar plates after 24 h incubation at 37°C, comparing GO@MA·TMA membrane to control samples. (b) Corresponding antibacterial rates of MA·TMA membrane and GO@MA·TMA membrane against *E. coli* and *S. aureus*. (c) Schematic illustration of the proposed antibacterial mechanism of GO@MA·TMA membrane. (d) HCHO removal efficiency of regular mask, N95 mask, MA·TMA membrane, and GO@MA·TMA membrane; the inset is the ESP mapping of HCHO. (e) Comparative heat dissipation performance of regular mask, N95 mask, MA·TMA membrane, and GO@MA·TMA membrane. (f) UV−vis−IR transmittance spectra of the MA·TMA membrane and GO@MA·TMA membrane. (g) Photograph of a UV indicator card demonstrating the comparative UV‐shielding performance of regular mask, N95 mask, MA·TMA membrane, and GO@MA·TMA membrane.

Airborne particulate pollutants are known vectors for diverse bacterial species, including pathogens that can induce allergic reactions and respiratory disorders, making the antimicrobial properties of filtration materials critical [48]. Accordingly, the antibacterial efficacy of the GO@MA·TMA membranes was evaluated against Gram‐negative *E. coli* (a predominant gastrointestinal pathogen) and Gram‐positive *S. aureus* (a leading cause of cutaneous and respiratory infections). As visualized in Figure 5a, while control groups supported vigorous bacterial proliferation, the pristine MA·TMA membrane exhibited only moderate inhibition (46.2% versus *E. coli*; 33.9% versus *S. aureus*). In striking contrast, the incorporation of GO nanosheets produced a dramatic enhancement, with the 0.5 wt.% GO@MA·TMA membrane achieving exceptional bacterial inhibition rates of 99.4% for *E. coli* and 99.8% for *S. aureus* (Figure 5b). This superior antibacterial performance is attributed to a synergistic dual‐action mechanism [49, 50, 51] imparted by GO nanosheets (Figure 5c). First, the atomically sharp edges of the 2D nanosheets function as “nano‐knives”, inducing severe membrane stress upon bacterial contact to disrupt cell integrity. Second, the oxidative stress mechanism was quantitatively validated by electron paramagnetic resonance (EPR) spectroscopy utilizing 5,5‐dimethyl‐1‐pyrroline‐N‐oxide (DMPO) as the spin‐trapping agent. Distinct characteristic signals corresponding to hydroxyl radicals (•OH, 1:2:2:1 quartet) and superoxide radicals (•O_2_^‒^, 1:1:1:1 quartet) were clearly detected in the GO@MA·TMA supramolecular system (Figure S13). These highly reactive oxygen species (ROS) cause irreversible oxidative damage to essential cellular components like lipids, proteins, and DNA, ultimately triggering bacterial apoptosis.

The membrane's capacity for removing hazardous VOCs was evaluated using HCHO—a pervasive indoor pollutant and classified carcinogen—as a model toxic gas. Utilizing a custom‐built filtration device (Figure S14), the optimized 0.5 wt.% GO@MA·TMA membrane achieved a 95% HCHO removal efficiency (Figure 5d; Figure S15), significantly outperforming control samples, including regular mask, N95 mask, and pristine MA·TMA membrane (Note: further quantitative analysis regarding the static adsorption capacity and cyclic regeneration performance is detailed in Figure S16). This high efficiency is attributed to a synergistic combination of physical and chemical capture mechanisms. First, the incorporation of GO nanosheets, which increased the specific surface area by 139% (from 12.09 to 28.88 m^2^ g^−1^) compared to the pristine membrane, provides a vastly expanded interface for the physical adsorption of HCHO molecules. Second, this high‐surface‐area framework is chemically active. As corroborated by the molecular electrostatic potential (ESP) mapping (Figures 4b and 5d, inset), the GO@MA·TMA supramolecular matrix exposes abundant high‐dipole functional groups (e.g., ─OH, ─NH_2_, ─COOH). These sites exhibit strong electrostatic complementarity with polar HCHO molecules, enabling efficient capture through multi‐point electrostatic attractions and hydrogen bonding, thereby firmly locking the pollutants within the membrane.

Wearer comfort is equally critical, and it is largely determined by the thermal management. Infrared thermographic analysis (Figure 5e) revealed that commercial N95 and regular masks maintained lower surface temperatures of 29.6°C and 31.1°C, respectively, indicating heat accumulation within the mask microclimate due to poor thermal conductivity. In contrast, the GO@MA·TMA membrane stabilized at 33.2°C, closely matching the skin temperature (33.4°C). This higher external surface temperature confirms the formation of an effective thermal transfer pathway, allowing metabolic heat to be rapidly conducted away from the face and dissipated via radiation and convection. This observation was directly corroborated by the UV–vis–IR spectra (Figure 5f), which showed that while the pristine MA·TMA membrane possessed some intrinsic transmittance in the infrared region [32], the incorporation of GO nanosheets significantly enhanced this property, particularly at wavelengths greater than 1300 nm. This enhanced infrared transmittance, especially in the short‐wave infrared range, contributes to the membrane's ability to regulate heat exchange. Intriguingly, this counter‐intuitive enhancement is a direct result of GO's role as a structural modulator [52]. The pristine MA·TMA membrane serves as a highly crystalline assembly, where long‐range molecular order and grain boundaries induce significant optical scattering and vibrational absorption. As confirmed by XRD, the incorporation of GO nanosheets disrupts this long‐range order, reducing the overall crystallinity. This structural disruption effectively attenuates these intrinsic scattering and absorption losses, widening an optical window that facilitates the transmission of infrared radiation, thereby allowing metabolic heat to radiate directly from the skin to the environment.

In addition to engineering the membrane for thermal comfort, the GO nanosheets integration also imparts potent UV‐shielding capabilities. The GO@MA·TMA membrane exhibited exceptional UV‐blocking efficacy, suppressing UV radiation transmittance (280–400 nm) to below 0.4% (Figure 5f). This was visually confirmed using UV‐detection cards, where the area covered by the GO@MA·TMA membrane showed the weakest color change (Figure 5g). This shielding capability originates from a dual mechanism: (i) the inherent UV absorption by GO's conjugated *π*‐structure and (ii) the reflection of UV radiation from its unique 2D planar structure [53]. Ultimately, this work demonstrates an optimized thermo‐photoprotective performance, wherein the membrane robustly blocks UV radiation while facilitating effective thermal management.

## 3 Conclusion

In summary, we have demonstrated the rational design of a multifunctional and fully recyclable GO@MA·TMA membrane via an environmentally benign, thermoreversible self‐assembly strategy based on the TIPC process. By leveraging GO nanosheets as heterogeneous nucleation‐templating agents, this approach enables tailored modulation of the supramolecular nanofibrous architecture, leading to a membrane with exceptional performance. The resulting composite membrane synergistically provides ultrahigh filtration efficiency (99.48% for PM_1.0_) and a comprehensive suite of functions, including broad‐spectrum antimicrobial activity (99.4% versus *E. coli* and 99.8% versus *S. aureus*), high‐efficiency HCHO removal (95%), effective thermal management for wearer comfort, and robust UV‐shielding (< 0.4% transmittance). Critically, the dynamic, thermoreversible nature of the supramolecular network facilitates a complete closed‐loop recycling of both MA·TMA nanocrystalline fibers and GO nanosheets. This work establishes a robust blueprint for designing next‐generation advanced materials that resolve the common trade‐off between high performance and lifecycle sustainability, opening a new avenue for high‐value applications in personal protective equipment and environmental purification systems.

## 4 Experimental Section

### 4.1 Materials

Melamine (MA, 99%) and trimesic acid (TMA, 98%) were purchased from Shanghai Aladdin Chemical Co. Graphene oxide dispersion was supplied from Suzhou Tanfeng Graphene Technology Co., Ltd. Ethanol (EtOH, 99.5%) and Formaldehyde (HCHO, 37%) were purchased from Sinopharm Chemical Reagent Co., Ltd. The *E. coli* and *S. aureus* were purchased from the Shanghai Bioresource Collection Center. Nutrient agar and nutrient broth were provided by Beijing Land Bridge Technology Co., Ltd. Phosphate buffer saline (PBS) was purchased from Beijing Solarbio Science and Technology Co., Ltd. All reagents were of analytical grade (AR) and used without further purification.

### 4.2 Synthesis of MA·TMA Supramolecular Precursor

In a typical synthesis, 0.8 g of MA (0.006 mol) and 1.33 g of TMA (0.006 mol) were dissolved in 40 mL of EtOH. The mixture was stirred at 60°C for 24 h, during which a white suspension formed. The resulting precipitate was collected by suction filtration, washed three times with EtOH, and subsequently dried in a vacuum oven at 60°C to yield the final MA·TMA supramolecular precursor as a white powder.

### 4.3 Preparation of GO@MA·TMA Membrane

The GO@MA·TMA membranes were prepared via a thermally induced precursor crystallization (TIPC) process. In a typical procedure, the previously synthesized MA·TMA precursor (0.03 wt.%) was dissolved in deionized water at 75°C with vigorous stirring to obtain a clear, homogeneous solution. Subsequently, a predetermined amount of GO dispersion was added to the heated precursor solution to achieve the target GO concentrations (0, 0.1, 0.5, 1.0, and 1.5 wt.% relative to the MA·TMA precursor). The mixture was then allowed to cool gradually to room temperature. During this cooling process, the supramolecular self‐assembly was initiated, leading to the formation of a 3D nanofibrous network. The resulting nanofiber suspension was collected by vacuum filtration and dried to yield a freestanding membrane, denoted as x wt.% GO@MA·TMA, where x is the mass percentage of GO.

### 4.4 Characterization

The morphologies of samples were examined using field‐emission scanning electron microscopy (FE‐SEM; Hitachi Regulus8100, Japan). Transmission electron microscopy (TEM) images and selected‐area electron diffraction (SAED) patterns of samples were obtained on a transmission electron microscope (TEM, JEOL JEM‐2100Plus, Japan). Fiber diameter distributions of samples were determined by measuring at least 80 randomly selected fibers from the SEM images using ImageJ software. Nitrogen adsorption‐desorption isotherms were measured at 77 K using a gas adsorption analyzer (Micromeritics 3Flex, USA) to determine the specific surface area and pore size distribution of samples. Crystallographic analysis was performed using an X‐ray diffractometer (XRD, Rigaku Ultima‐IV, Japan) with Cu Kα radiation (λ = 1.5418 Å). Chemical structures of samples were analyzed by Fourier‐transform infrared (FTIR) spectroscopy (Nicolet iS10, Thermo Scientific, USA) and Raman spectroscopy (ThermoFisher DXR2, USA). Thermal stability of samples was evaluated using a thermogravimetric analyzer (TGA, Mettler Toledo TGA/DSC 3+, Switzerland). Electron paramagnetic resonance spectra were obtained using an electron paramagnetic resonance spectrometer (EPR, EMX plus, Bruker, Germany). The optical transmittance spectra of samples were recorded on a UV–vis–NIR spectrophotometer (Shimadzu UV‐3600i Plus, Japan) equipped with an integrating sphere.

### 4.5 Density Functional Theory Calculations

All DFT calculations were performed using the Vienna Ab initio Simulation Package (VASP) with the Perdew–Burke–Ernzerhof (PBE) functional within the generalized gradient approximation (GGA). The projected augmented wave (PAW) method was employed to describe the ionic cores, with valence electrons represented by a plane‐wave basis set using a kinetic energy cutoff of 450 eV. We applied Gaussian smearing with a width of 0.05 eV for partial occupancies of the Kohn‐Sham orbitals. Geometry optimizations and lattice parameter determinations were conducted using a *Γ*‐centered 1 × 2 × 1 k‐point mesh for Brillouin zone integration, with convergence criteria of 10^−5^ eV for electronic self‐consistency and 0.02 eV Å^−1^ for atomic forces. To minimize periodic boundary effects, a 15 Å vacuum layer was introduced. van der Waals interactions were accounted for using the DFT‐D3 empirical correction scheme, and spin polarization was included for magnetic systems. Charge density difference analysis was performed using VASPKIT (version 1.3.5).

The free energy (ΔG) for each reaction step was calculated as:

(1) ΔG=ΔEZPE+ΔE−TΔS

where Δ*EZPE* represents the zero‐point energy at 298.15 K, Δ*E* is the binding energy of intermediates, *T* is the temperature (298.15 K), and Δ*S* denotes the entropy change.

### 4.6 Filtration Performance

The PM removal efficiency and associated pressure drop of the GO@MA‐TMA membrane were evaluated using an air filtration tester (Model‐1, Pansitech, China). To simulate heavily polluted air with high PM (PM_2.5_> 2000 µg m^−3^, PM_10_> 4000 µg m^−3^) concentrations, cigarette smoke was utilized as the source, which contains abundant particulate matter and organic pollutants such as nicotine and polycyclic aromatic hydrocarbons (PAHs). The filtration efficiency (E_C_, %) was calculated using the following equation:

(2) $$E_{C}=\frac{C_{0}-C}{C_{0}}\;\;\times \;100\%$$

where *C_0_* and *C* represent the PM concentrations (particles/cm^3^) upstream and downstream of the filtration membrane, respectively. The filtration performance was further quantified by calculating the quality factor (QF, Pa^−^^1^) according to the standard metric:

(3) $$\mathrm{QF}\;=\frac{-\ln\left(1-E\right)}{\Delta P}\;$$

where *E* is the removal efficiency for PM, and *∆P* is the pressure drop before and after samples (Pa).

### 4.7 Outdoor Air Filtration Test

The practical air filtration performance of the membrane was evaluated via an outdoor field test conducted on a university campus in Qingdao, China (June 5–15, 2025). A custom‐built sampling apparatus was deployed outdoors to record PM concentrations at three fixed time points daily (09:00–15:00, Beijing Standard Time). This testing period encompassed a range of real‐world atmospheric conditions, including both clear and hazy days, to assess the membrane's stability and reliability.

### 4.8 Antibacterial Test

The antibacterial efficacy of the membranes was quantitatively evaluated against *E. coli* and *S. aureus* using the colony‐forming unit (CFU) counting method, following the Chinese National Standard GB/T 20944.3‐2008. The antibacterial ratio (R, %) was calculated by comparing the number of viable bacterial colonies after incubation with the test samples versus the control samples.

### 4.9 Formaldehyde Removal Performance Test

The formaldehyde (HCHO) removal performance of the membranes was evaluated in a sealed chamber. An aqueous HCHO solution (1:50 v/v in deionized water) was atomized into the chamber and allowed to fully volatilize. The initial HCHO concentration for the test was stabilized at 4.0 ± 0.2 ppm under controlled environmental conditions (25 ± 1°C and 50 ± 5% RH). The HCHO concentration was monitored over time using colorimetric GASTEC detector tubes (171SC, 0.5–4 ppm detection range, Japan) to determine the removal efficiency. HCHO static adsorption performance was evaluated using a custom‐built static test system comprising a sealed 3 L acrylic chamber and a portable HCHO detector (HTV‐M, PPM Technology, UK). To establish a stable test atmosphere, a 1% formaldehyde solution was vaporized within the chamber until the concentration reached approximately 10 ppm, after which the liquid source was removed. The chamber was then sealed and equilibrated for 6 h to ensure gas homogeneity. Subsequently, the membrane sample was introduced, and the HCHO concentration was monitored under static conditions at intervals of 0, 2, and 24 h.

### 4.10 Statistical Analysis

All experiments were conducted with at least three parallel replicates, and the experimental data were presented as mean ± standard deviation (SD). All data processing was performed using Origin software (OriginLab, USA).

## Funding

The authors acknowledge the financial support from the Qingdao Natural Science Foundation (Grant no. 23‐2‐1‐16‐zyyd‐jch), Qingdao Postdoctoral Program (Grant no. QDBSH20230101002), and Medium‐sized Enterprises Innovation Capacity Enhancement Project of Shandong Province (Grant no. 2024TSGC0692).

## Conflicts of Interest

The authors declare no conflicts of interest.

## Supporting information




**Supporting File**: advs74854‐sup‐0001‐SuppMat.pdf.

## Data Availability

The data that support the findings of this study are available from the corresponding author upon reasonable request.
