# Supplementary material for: A High‐Performance and Fully Recyclable Supramolecular Nanofibrous Membrane for Multifunctional Air Filtration
Source: Adv Sci (Weinh). 2026 Mar 18;13(30):e23204. doi: 10.1002/advs.202523204 (PMC13248783; doi:10.1002/advs.202523204)
Supplement: Supplementary file 1 — Supporting File: advs74854‐sup‐0001‐SuppMat.pdf. [file ADVS-13-e23204-s001.pdf]

Supporting Information

**A High-Performance and Fully Recyclable Supramolecular Nanofibrous Membrane for Multifunctional Air Filtration**

*Wenjing Sun, Ding Yuan*, Jiaqi Wan, Junyu Chen, Zhen Yan, Yinuo Yan, Senjie Dong**

Industrial Research Institute of Nonwovens &Technical Textiles, Shandong Engineering Research Center for Specialty Nonwoven Materials, College of Textiles & Clothing, Qingdao University, Qingdao 266071, People's Republic of China

*Corresponding authors.

E-mail: yuanding@qdu.edu.cn, senjiedong@qdu.edu.cn

This file includes:

Figures S1 to S16

Tables S1 to S2

Notes S1 to S2

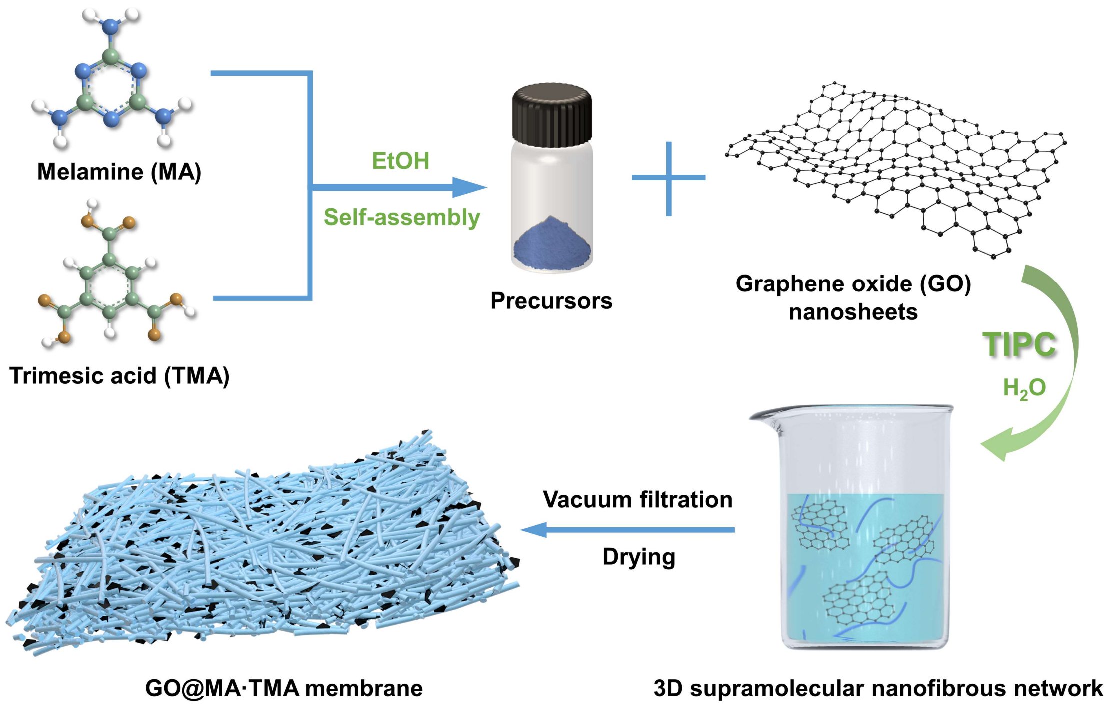


**Figure S1**. Schematic illustration of the preparation process of GO@MA·TMA membrane. The two-stage process involves: (i) the initial self-assembly of MA and TMA in ethanol to form the supramolecular precursors, followed by (ii) the TIPC of precursors in an aqueous solution containing dispersed GO nanosheets, leading to the formation of the final nanofibrous membrane upon drying.

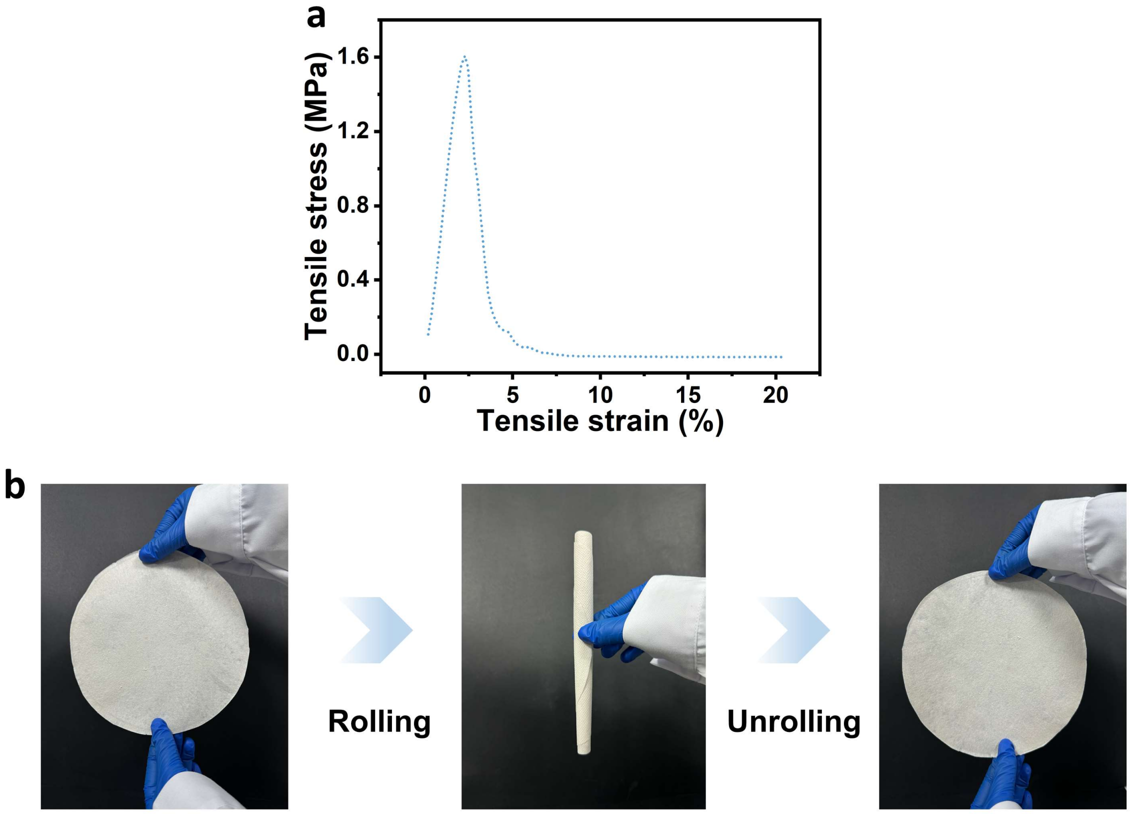


**Figure S2.** Mechanical characterization and flexibility of the GO@MA·TMA membrane. (a) The typical stress-strain curve of the composite membrane, from which a tensile strength of 1.6 ± 0.2 MPa and an elongation at break of 2.3 ± 0.3% were derived. (b) Digital images illustrating the excellent flexibility and structural integrity of the composite membrane during rolling.

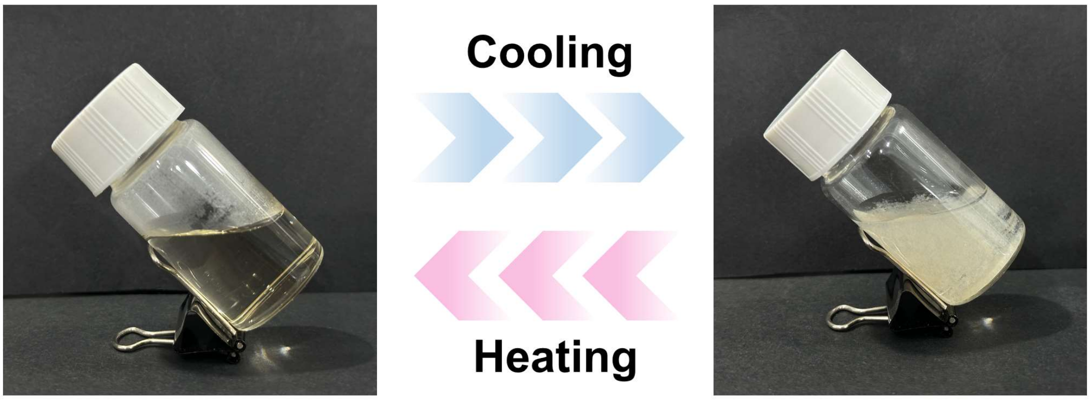


**Figure S3.** Illustration of the thermoreversible phase transition of the GO@MA·TMA supramolecular system. The figure demonstrates the reversible cycle where the fully formed GO@MA·TMA 3D supramolecular nanofibrous network (solid phase) completely dissolves into its constituent precursors upon heating, and the network spontaneously re-formed via a TIPC process upon cooling. This equilibrium-driven behavior is the fundamental basis for the GO@MA·TMA membrane's closed-loop recyclability.

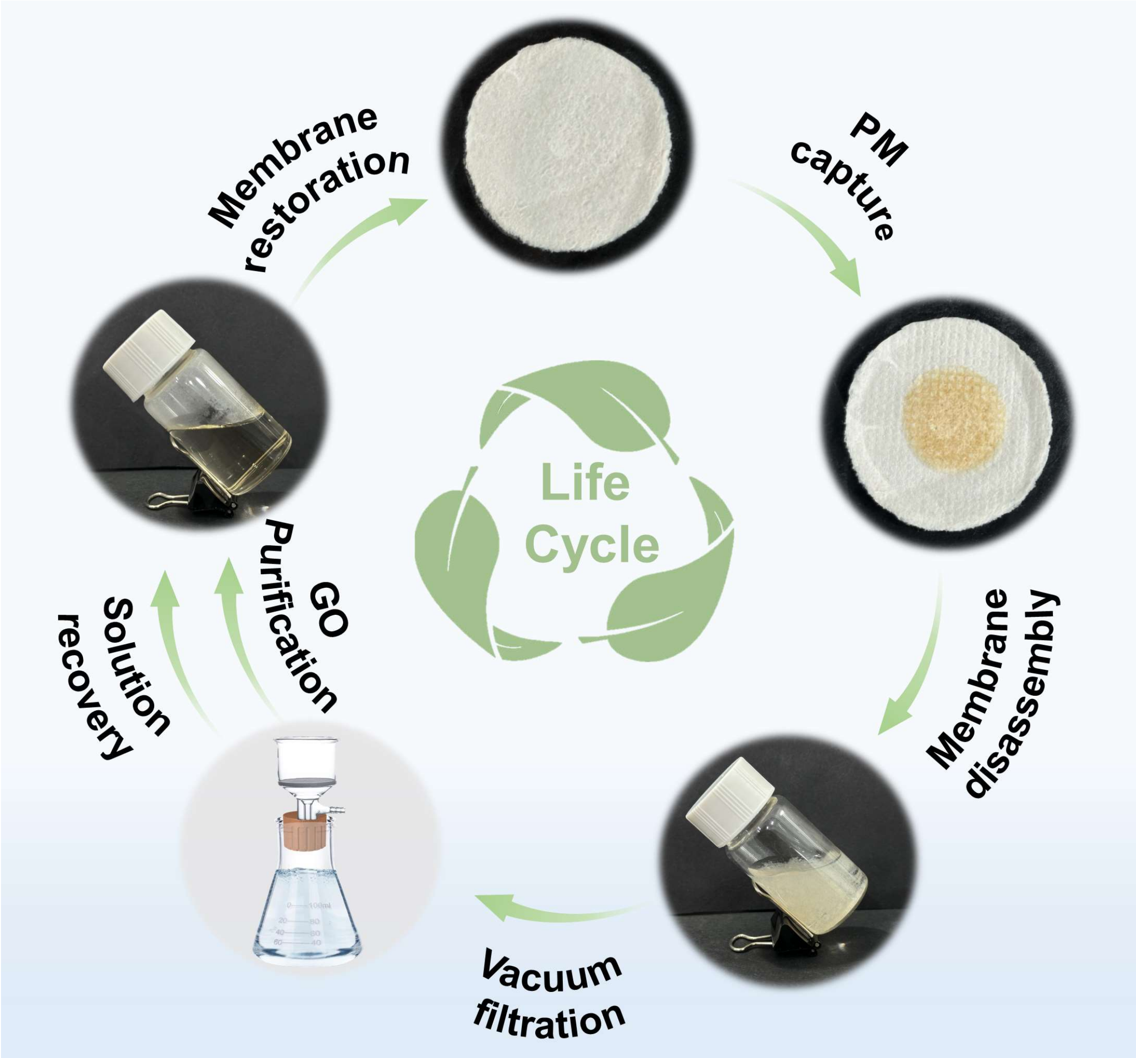


**Figure S4.** Illustration of the closed-loop recycling protocol for the GO@MA·TMA membrane. The process involves three main stages: (i) Thermal dissolution and purification, where used composite membranes are dissolved by heating to separate the MA·TMA precursor saturated solution from solid contaminants (composed of GO nanosheets and oily PM); (ii) GO recovery, where the captured GO nanosheets are purified through a combined ultrasonic-centrifugal cleaning process with a recovery efficiency of 91%; and (iii) Membrane restoration, where the MA·TMA precursor saturated solution and recovered GO are recombined to regenerate the composite membranes.

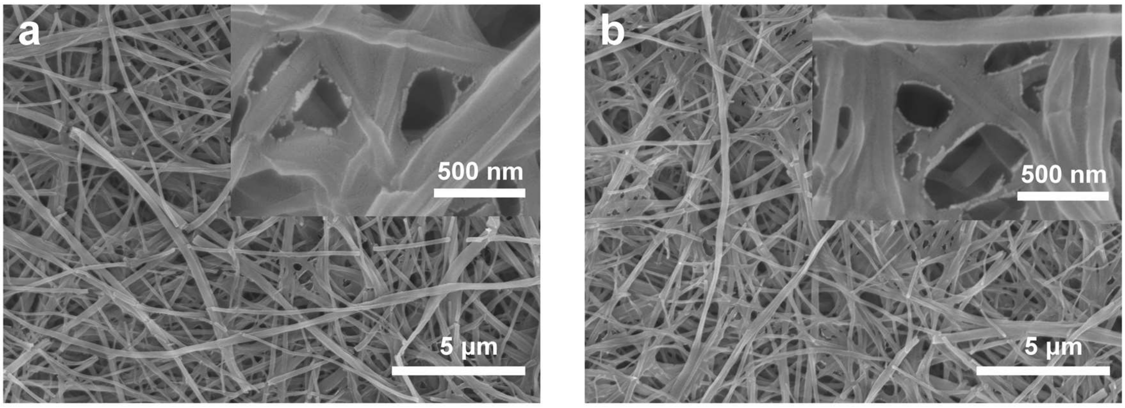


**Figure S5.** Validation of microstructural integrity after the closed-loop recycling process. Comparative SEM images of the 0.5 wt% GO@MA·TMA membrane (a) before (pristine) and (b) after one cycle of dissolution and reconstitution (recovered). These images confirm that the interconnected, nanofibrous architecture is fully retained after recycling, with no discernible morphological degradation.

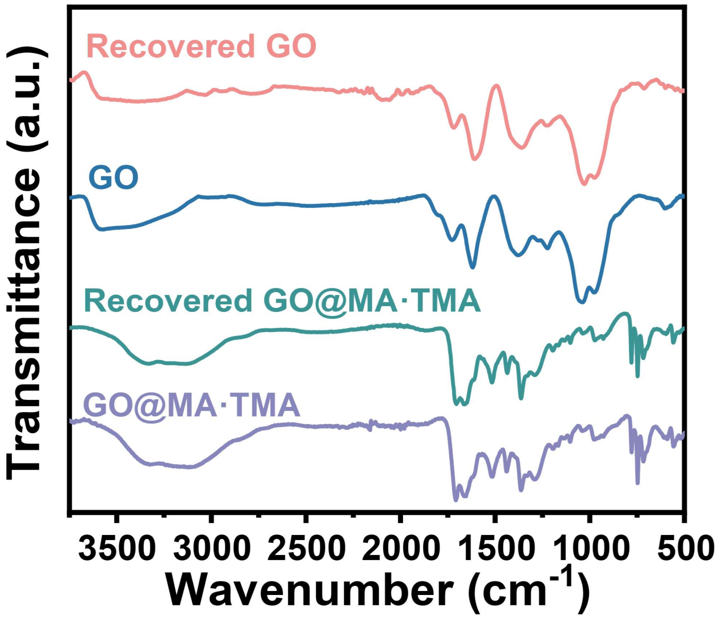


**Figure S6.** Validation of chemical integrity after the closed-loop recycling process. This figure present a comparative FTIR analysis of: (i) the pristine versus the recovered GO nanosheets, and (ii) the pristine versus the recovered GO@MA·TMA membrane. The near-identical nature of the corresponding spectra confirms that the characteristic molecular signatures and functional groups of both individual GO component and composite membrane remain fully intact after the recycling protocol.

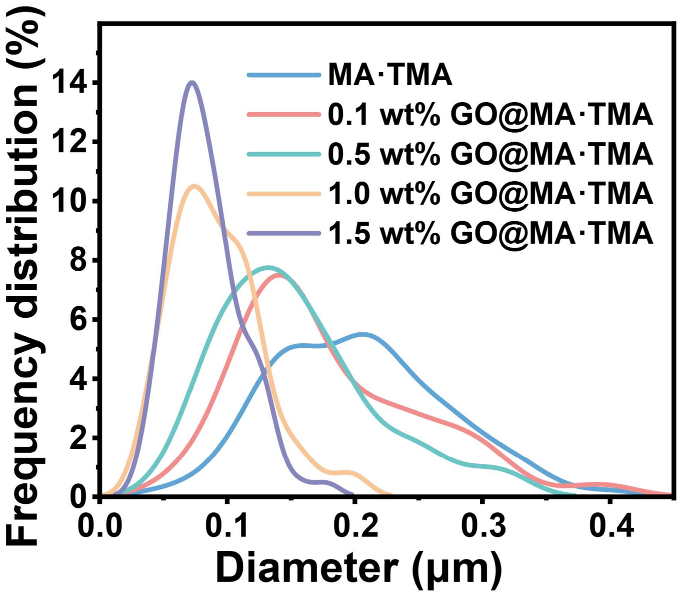


**Figure S7.** Fiber diameter distributions for GO@MA·TMA membranes with varying GO concentrations. The curves clearly show a progressive shift toward smaller average fiber diameters and a narrower size distribution as the GO content increases, confirming the role of GO nanosheets in modulating the nanofiber architecture.

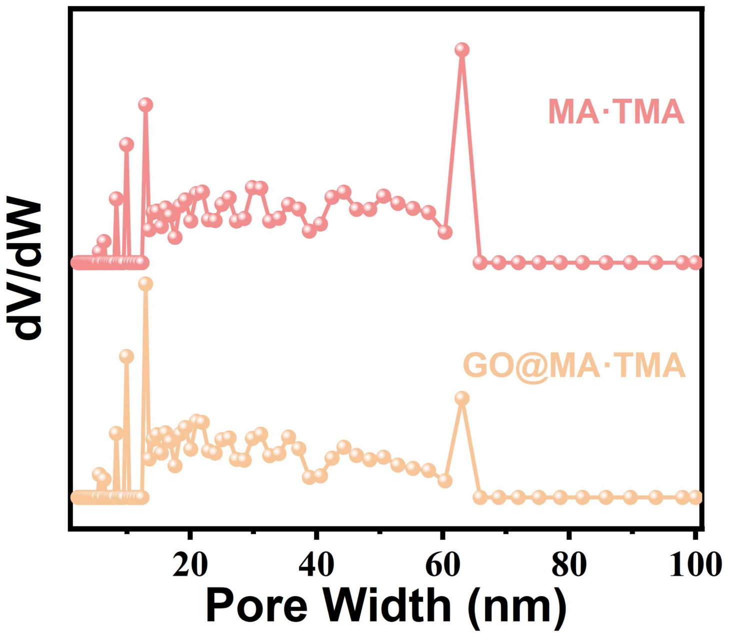


**Figure S8.** Pore size distribution of the pristine MA·TMA membrane versus the GO@MA·TMA membrane. The results show that the incorporation of GO nanosheets significantly refines the pore structure, leading to a substantial increase in the proportion of small mesopores (e.g., < 20 nm). This optimized pore architecture is critical for enhancing the composite membrane's PM capture efficiency.

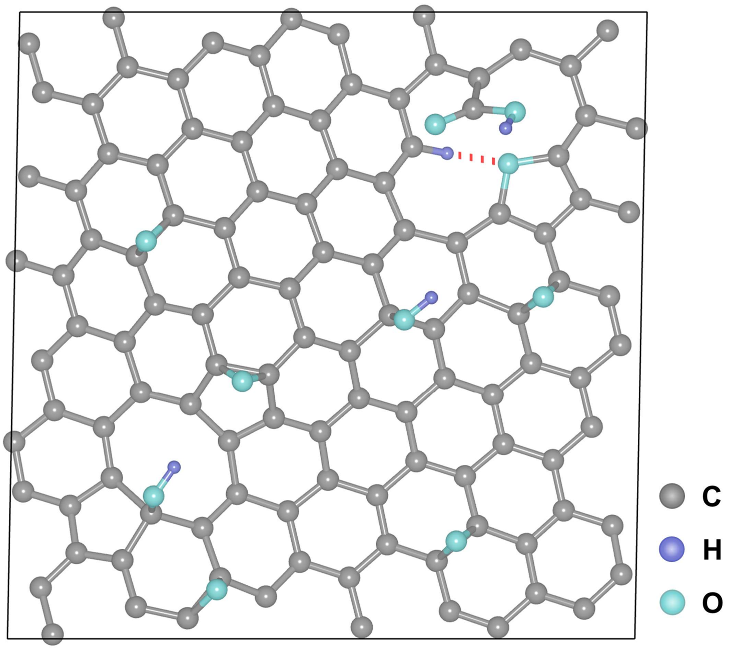


**Figure S9.** Structural model of the GO framework employed for the DFT calculations. The model depicts a representative section of a GO nanosheet, featuring both the sp2 graphitic lattice and various key oxygen-containing functional groups, including hydroxyl (-OH), epoxy (-COC), and carboxyl (-COOH) groups, which serves as the active sites for molecular adsorption.

**Note S1.** Discussion on the limitations of the idealized DFT model regarding real GO defects.

It is important to note that the DFT models employed here represent an idealized GO structure with uniform functional group distribution. In experimental reality, GO nanosheets are "defect-rich", containing vacancies, edges, and irregular oxidation zones. However, these structural defects typically possess higher surface energies and may serve as even more potent active sites for adsorbing MA/TMA molecules than the defect-free regions simulated here.[1] Therefore, the reduced nucleation barriers calculated in this work should be viewed as a conservative estimate; the actual heterogeneous nucleation effect on real GO surfaces is likely more pronounced, further validating the mechanism.

**Note S2.** Theoretical correlation between nucleation kinetics and fiber diameter based on classical nucleation theory (CNT).

To quantitatively correlate the DFT-calculated energetic advantage with the experimentally observed fiber refinement, we apply the CNT.[2] The nucleation rate (J) is governed by the energy barrier ($\Delta G$) according to:

$$J = A\,exp\left(-\frac{\triangle G}{k_B T}\right) \qquad (1)$$

where A is the pre-exponential factor. The substantial reduction in ΔG on the GO surface implies an exponential increase in J, leading to a significantly higher nucleation density (N). Given a fixed total mass of precursors ($M_{total}$), the final mass of each individual fiber ($m_{fiber}$) is inversely proportional to the nucleation density:

$$m_{fiber} = \frac{M_{total}}{N} \qquad (2)$$

Assuming the fibers are cylindrical with density ρ and length L, the fiber diameter (D) scales as:

$$\mathrm{D} \propto \sqrt{\frac{m_{fiber}}{\rho \mathrm{L}}} \propto \frac{1}{\sqrt{N}} \qquad (3)$$

This relationship explicitly indicates that the proliferation of nucleation sites (N↑) triggered by GO inevitably forces the distribution of solute into a larger number of finer fibers (D↓). This theoretical derivation aligns perfectly with our SEM observations, where the fiber diameter decreased from 204 to 84 nm as the GO-induced nucleation density increased.

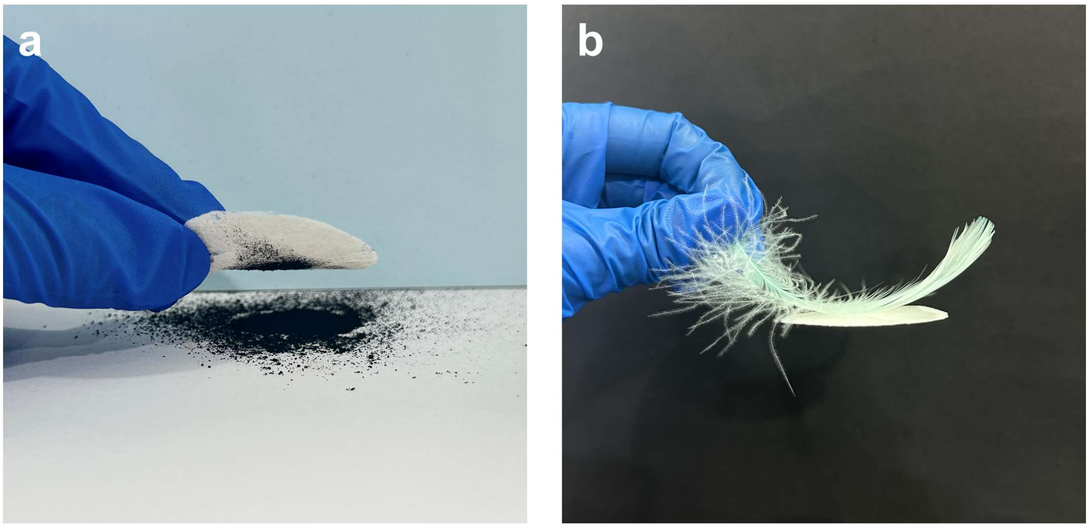


**Figure S10.** Macro-scale visual demonstration of the potent electrostatic effect of the GO@MA·TMA membrane. The digital images show that the membrane can attract and firmly adsorb lightweight, insulating objects like (a) carbon powder and (b) a feather.

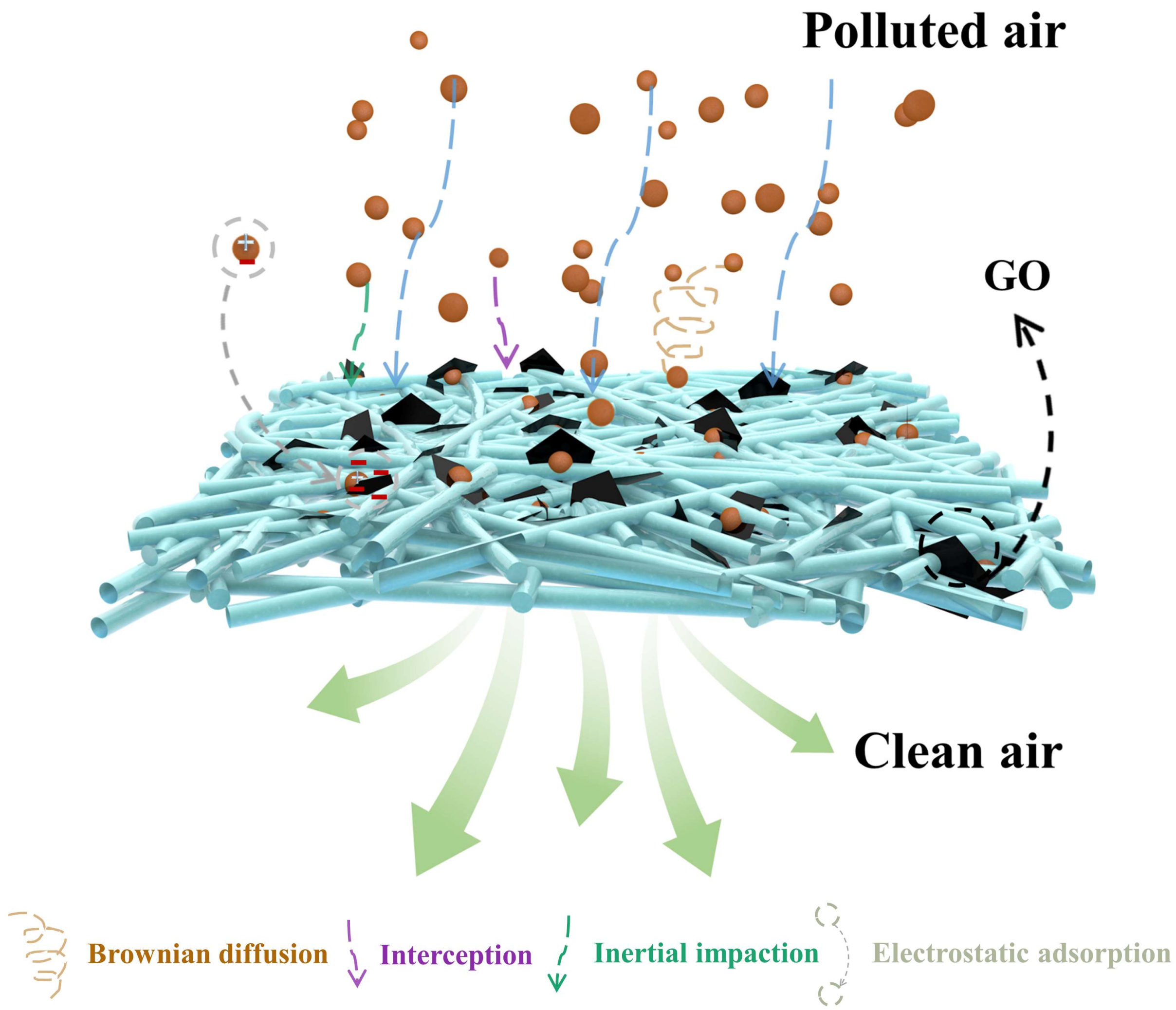


**Figure S11.** Schematic illustration of the synergistic PM capture mechanisms in the GO@MA·TMA membrane. The membrane's high filtration efficiency originates from a dual-mode action: (i) Enhanced passive mechanical filtration, driven by the optimized nanofibrous network structure, which facilitates physical interception, inertial impaction, and Brownian diffusion of particles; and (ii) Active electrostatic adsorption, primarily attributed to the integrated GO nanosheets which create highly polar surfaces and net surface charges that strongly attract ultrafine PM.

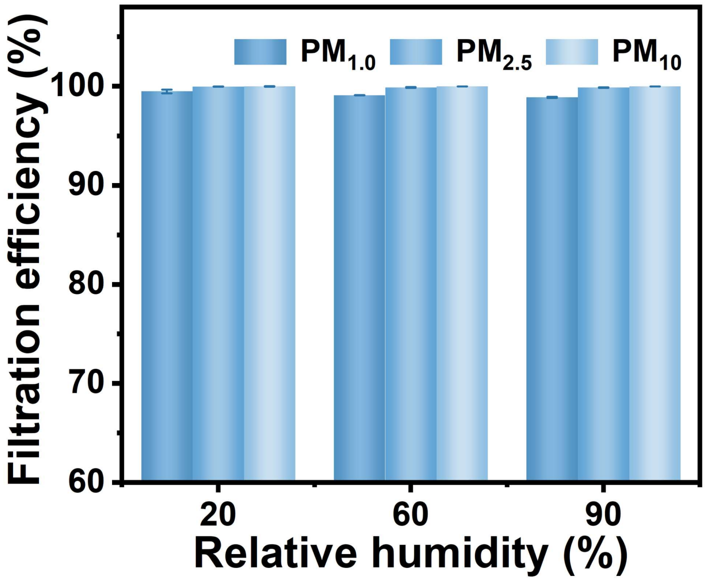


**Figure S12.** Evaluation of the filtration stability of the 0.5 wt% GO@MA·TMA membrane under varying relative humidity (RH). The plot showed that the composite membrane maintains exceptionally high and stable filtration efficiency for all PM sizes, even when the RH was increased to 90%. This demonstrates the composite membrane's robust performance and reliability in humid environments.

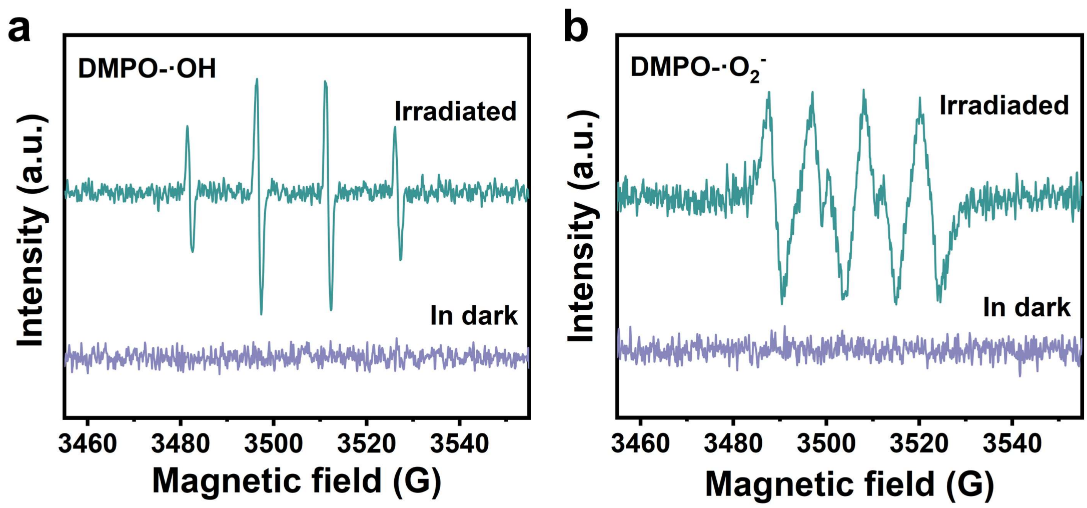


**Figure S13.** Spectroscopic validation of reactive oxygen species (ROS) generation. Typical electron paramagnetic resonance (EPR) spectra of DMPO spin-trapping adducts detected in the GO@MA·TMA supramolecular system. The observation of intense characteristic peaks for hydroxyl radicals (•OH, 1:2:2:1 quartet) and superoxide radicals (•$O_2^-$, 1:1:1:1 quartet) confirmed the chemical mechanism of oxidative stress mediated by the GO nanosheets. These signals provide direct experimental evidence that the GO@MA·TMA membrane effectively generates lethal ROS upon photo-excitation.

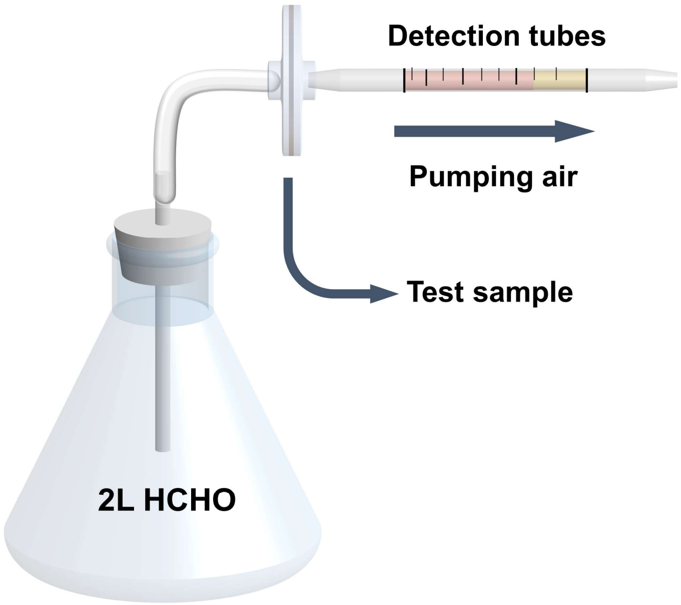


**Figure S14.** Schematic of the experimental setup used to measure formaldehyde (HCHO) removal efficiency of GO@MA·TMA membrane. In this custom-built device, HCHO gas is pumped from a 2L source container through the test sample. The downstream concentration of HCHO in the filtered air is then quantified using colorimetric detection tubes.

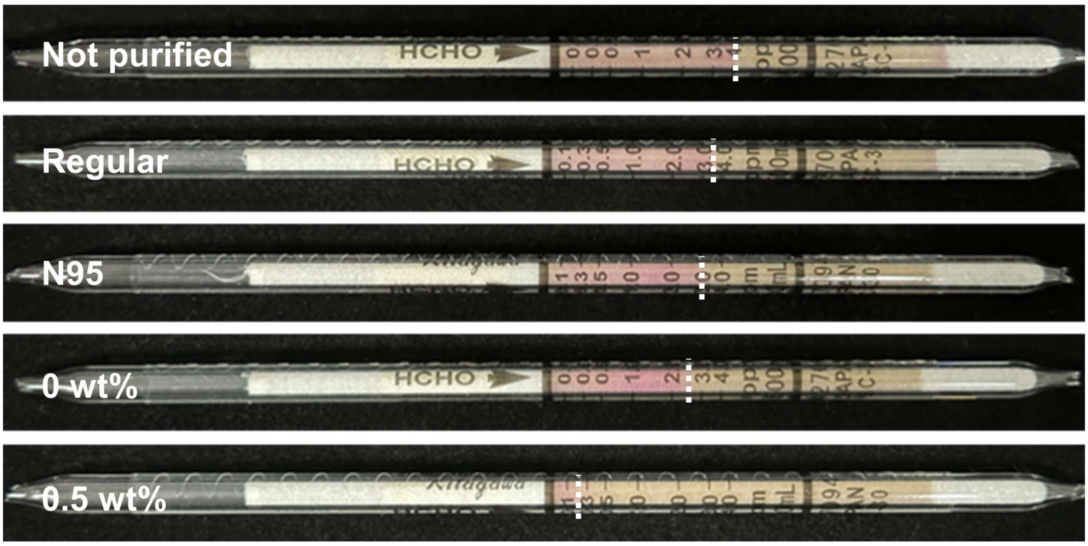


**Figure S15.** Visual evidence for the high-efficiency removal of HCHO by the GO@MA·TMA membrane compared to control filters. The photographs showed the colorimetric readings of HCHO detector tubes for different samples. The "Not purified" tube indicated a high initial HCHO concentration before filtration. The regular mask demonstrated negligible HCHO removal, with stain lengths comparable to the initial concentration. The N95 mask and the pristine MA·TMA membrane showed moderate adsorption capability. In stark contrast, the tube corresponding to the 0.5 wt% GO@MA·TMA membrane exhibited minimal color change, signifying near-complete HCHO removal from the gas stream. This direct visual comparison confirms composite membrane's potent VOCs adsorption capability.

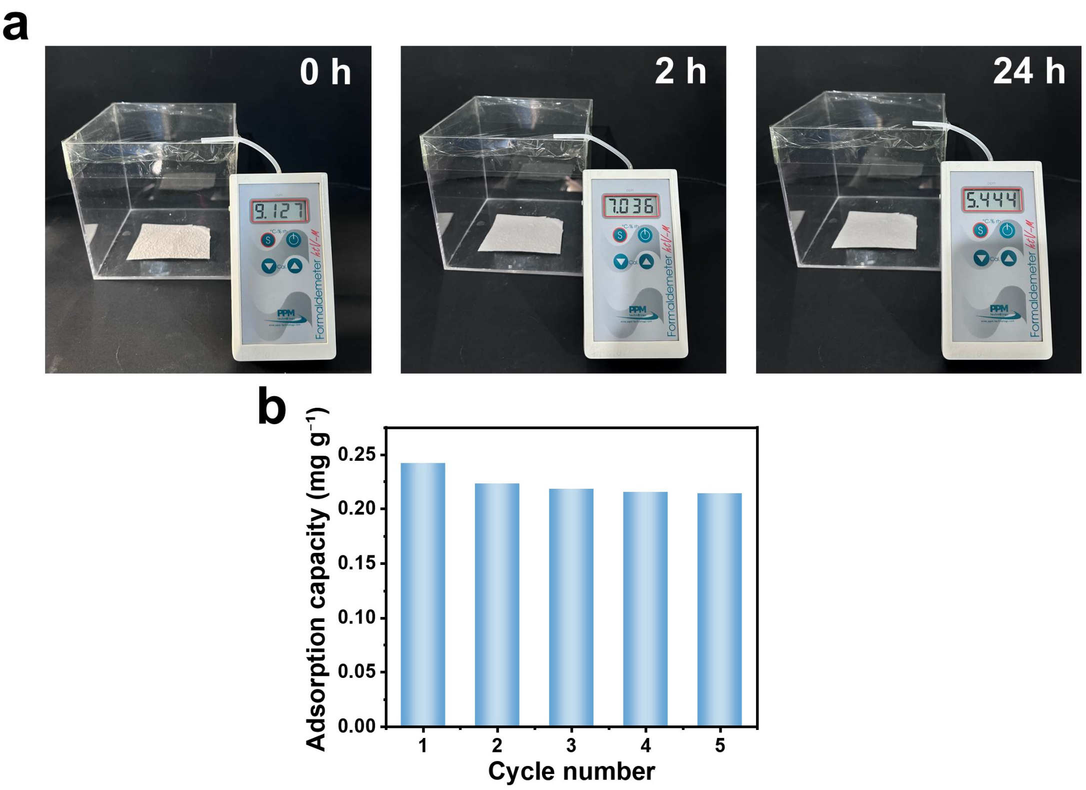


**Figure S16.** HCHO quantitative adsorption performance and regenerability of the GO@MA·TMA membrane. (a) Static adsorption capacity evaluated in a 3 L sealed chamber with an initial HCHO concentration of ~10 ppm. The membrane achieved an equilibrium capacity of 0.242 mg $g^{-1}$. (b) Cyclic stability test over five consecutive adsorption–desorption cycles. The HCHO-saturated membrane was regenerated via thermal vacuum treatment at 60 °C. The slight capacity decay (retaining 88.4% at the 5th cycle) is attributed to the irreversible chemisorption on high-energy active sites.

**Tables S1**

Air filtration performance compared between the GO@MA·TMA membrane and other air filters.

| Materials | Airflow velocity (cm $s^{-1}$) | Particulate matters (μm) | Filtration efficiency (%) | Pressure drop (Pa) | Quality factor ($Pa^{-1}$) | Reference |
|---|---|---|---|---|---|---|
| 0.5 wt% GO@MA·TMA | 5.3 | 2.5, burning cigarette | 99.96 | 97 | 0.082 | This work |
| Int-MN4.5 | 5.3 | 2.5, burning paper pieces | 93.47 | 37 | 0.074 | [3] |
| CS/PVA | 5.3 | 2.5, NaCl | 95.59 | 633 | 0.049 | [4] |
| P20 | 5.3 | 2.5, combusted incense | 94.35 | 119 | 0.025 | [5] |
| HAP/CT | 5 | 2.5, burning incense | 96.08 | 128 | 0.025 | [6] |
| PLA/LIG | 5.3 | 3, burning incense | 99.6 | 105 | 0.053 | [7] |
| PNWLFs | 4.16 | 2.5, NaCl | 99.76 | 106 | 0.055 | [8] |
| AcNBC | 4 | 2.5, burning incense | 97 | 300 | 0.011 | [9] |
| PTFE-Nylon (TAF) | 4 | 2.5, burning cigarette | 96 | 104 | 0.018 | [10] |
| 0.5 wt% GO@MA·TMA | 2.7 | 2.5, burning cigarette | 99.99 | 57 | 0.162 | This work |
| SS-CMPs-M | 2.4 | 2.5, burning sandalwood | 99.7 | 550 | 0.013 | [11] |
| PMIA/$SiO_2$-NF | 0.66 | 2.5, cigarette smoke | 97.33 | 125 | 0.028 | [12] |
| 0.5 wt% GO@MA·TMA | 10.6 | 2.5, cigarette smoke | 99.54 | 166 | 0.032 | This work |
| PA4-3 | 10.6 | 2.5, burning moxa stick | 94.36 | 118.3 | 0.024 | [13] |
| CNT aerogel | 10 | 0.006-2.5, dioctyl sebacate | 99 | 1946 | 0.004 | [14] |
| 0.5 wt% GO@MA·TMA | 12.3 | 2.5, cigarette smoke | 98.83 | 241 | 0.018 | This work |
| TPTF | 12 | 2.5, burning sandalwood | 96.5 | 91 | 0.041 | [15] |
| PI-ZIF8 | 14.4 | 2.5, cigarette smoke | 96.6 | 72 | 0.047 | [16] |

| | | | | | | |
|---|---|---|---|---|---|---|
| 0.5 wt% GO@MA·TMA | 5.3 | 1, cigarette smoke | 99.48 | 97 | 0.054 | This work |
| Int-MN4.5 | 5.3 | 1, burning paper pieces | 91.71 | 37 | 0.067 | [3] |
| VIO-NF | 5.3 | 1, dioctyl sebacate | 99.7 | 79 | 0.073 | [17] |
| AcNBC | 4 | 1, burning incense | 97.5 | 300 | 0.012 | [9] |

Note: Direct comparison of filtration performance is approximate due to variations in testing standards across studies, including aerosol types, face velocities, and detection methods. These values are presented for qualitative benchmarking purposes only.

**Tables S2**

Comparison of recycling strategies and performance retention between the GO@MA·TMA membrane and other recyclable air filters.

| Materials | Target Pollutant | Recycling Strategy | Efficiency Retention | Pressure Drop Change | Reference |
|---|---|---|---|---|---|
| 0.5 wt% GO@MA·TMA | $PM_{1.0, 2.5, 10}$ | Solution-reconstitution | >99.84% | 7.2% | This work |
| 1 wt% $SiO_2$@MA·TMA | $PM_{1.0, 2.5, 10}$ | Solution-reconstitution | >97.18% | 11.76% | [18] |
| 1 wt% COF@MA-TMA | $PM_{1.0, 2.5, 10}$ | Solution-reconstitution | ~100% | 1.1% | [19] |
| ATCAF | $PM_{2.5}$ | Heat-cleaning | ~100% | \ | [20] |
| PLLA | $PM_{1.0, 2.5}$ | Autoclaving, Microwaving, Washing | ~100% | <5.5% | [21] |
| F-PVDF-HFP/$SiO_2$ | $PM_{0.3}$ | Spray cleaning | ~100% | >8.13% | [22] |
| SBS/PAN | $PM_{0.3}$ | Water and ethanol washing | ~90% | ~90% | [23] |
| PP@ZIF-8/ PP@Cu-BTC | $PM_{1.0}$ | Solvent washing | >74.52% | No change | [24] |
| MSM-PLA | $PM_{0.3}$ | Water/IPA washing | >91.37% | \ | [25] |
| PTFE | $PM_{2.5}$ | Blowback Water droplet charging/cleaning | ~100% | <7% | [26] |

**References**


[1] a) B. Sanyal, O. Eriksson, U. Jansson, H. Grennberg, *Physical Review B* **2009**, 79, 113409; b) P. Lazar, F. Karlický, P. Jurečka, M. Kocman, E. Otyepková, K. Šafářová, M. Otyepka, *Journal of the American Chemical Society* **2013**, 135, 6372; c) N. Dimakis, F. A. Flor, A. Salgado, K. Adjibi, S. Vargas, J. Saenz, *Applied Surface Science* **2017**, 421, 252.

[2] a) S. Prestipino, A. Laio, E. Tosatti, *Physical Review Letters* **2012**, 108, 225701; b) V. I. Kalikmanov, in *Nucleation Theory*, DOI: 10.1007/978-90-481-3643-8_3 (Ed: V. I. Kalikmanov), Springer Netherlands, Dordrecht **2013**, p. 17.

[3] S. Choi, H. Jeon, M. Jang, H. Kim, G. Shin, J. M. Koo, M. Lee, H. K. Sung, Y. Eom, H.-S. Yang, J. Jegal, J. Park, D. X. Oh, S. Y. Hwang, *Advanced Science* **2021**, 8, 2003155.

[4] Z. Wang, F. Yan, H. Pei, J. Li, Z. Cui, B. He, *Carbohydrate Polymers* **2018**, 198, 241.

[5] R. Amalia, A. Noviyanto, L. A. Rahma, Merita, A. Labanni, M. Fahroji, S. Purwajanti, D. A. Hapidin, A. Zulfi, *Sustainable Materials and Technologies* **2024**, 40, e00928.

[6] Z.-C. Xiong, R.-L. Yang, Y.-J. Zhu, F.-F. Chen, L.-Y. Dong, *Journal of Materials Chemistry A* **2017**, 5, 17482.

[7] R. Scaffaro, M. C. Citarrella, *Sustainable Materials and Technologies* **2024**, 42, e01146.

[8] D. Shu, L. Wang, X. Liu, S. Luo, Y. Zhang, C. Cheng, Z. Li, L. Jiao, S. Yang, C. Li, L. Xia, P. Xi, B. Cheng, *Separation and Purification Technology* **2025**, 359, 130401.

[9] B. Sun, J. Lin, M. Liu, W. Li, L. Yang, L. Zhang, C. Chen, D. Sun, *ACS Sustainable Chemistry & Engineering* **2022**, 10, 1644.

[10] Y. Bai, C. B. Han, C. He, G. Q. Gu, J. H. Nie, J. J. Shao, T. X. Xiao, C. R. Deng, Z. L. Wang, *Advanced Functional Materials* **2018**, 28, 1706680.

[11] Y. Wang, Y. Su, L. Yang, M. Su, Y. Niu, Y. Liu, H. Sun, Z. Zhu, W. Liang, A. Li, *Journal of Membrane Science* **2022**, 659, 120728.

[12] L. Zhong, T. Wang, L. Liu, W. Du, S. Wang, *Separation and Purification Technology* **2018**, 202, 357.

[13] F. Zhan, Z. Wang, J. Shi, D. Zhang, M. Wang, Z. Yi, L. Zhao, *ACS Applied Polymer Materials* **2024**, 6, 1215.

[14] L. Issman, B. Graves, J. Terrones, M. Hosmillo, R. Qiao, M. Glerum, S. Yeshurun, M. Pick, I. Goodfellow, J. Elliott, A. Boies, *Carbon* **2021**, 183, 232.

[15] Z. Ding, Z. Tian, X. Ji, G. Yang, M. Sameer, Y. Lu, O. J. Rojas, *Advanced Functional Materials* **2024**, 34, 2313790.

[16] Z. Hao, J. Wu, C. Wang, J. Liu, *ACS Applied Materials & Interfaces* **2019**, 11, 11904.

[17] J. Lee, J. Bae, D.-Y. Youn, J. Ahn, W.-T. Hwang, H. Bae, P. K. Bae, I.-D. Kim, *Chemical Engineering Journal* **2022**, 444, 136460.

[18] W. Sun, S. Dong, M. Gao, H. Diao, Y. Song, L. Zhang, H. Wang, D. Yuan, *Macromolecular Rapid Communications* **2025**, 46, 2401019.

[19] D. Yuan, W. Sun, J. Wan, J. Chen, M. Gao, S. Dong, *Chemical Communications* **2025**, 61, 14685.

[20] X. Ji, J. Zhao, S. M. Jung, A. I. H. Hrdina, M. J. Wolf, X. Yang, G. Vaartstra, H. Xie, S. L. Luo, A. Y. Lu, R. E. Welsch, E. N. Wang, L. J. Li, J. Kong, *Nano Lett* **2021**, 21, 8160.

[21] T. T. Le, E. J. Curry, T. Vinikoor, R. Das, Y. Liu, D. Sheets, K. T. M. Tran, C. J. Hawxhurst, J. F. Stevens, J. N. Hancock, O. R. Bilal, L. M. Shor, T. D. Nguyen, *Advanced Functional Materials* **2022**, 32, 2113040.

[22] J.-P. Chen, C.-Y. Guo, Q.-J. Zhang, X.-Q. Wu, L.-B. Zhong, Y.-M. Zheng, *Journal of Membrane Science* **2023**, 675, 121545.

[23] Y. Gou, Y. Yang, W. Zheng, X. Ji, N. Lu, W. Wang, M. Zhong, Y. Shi, J. Huang, W. Cai, Y. Lai, *Environmental Science & Technology* **2024**, 58, 17376.

[24] Y. Cheng, W. Wang, R. Yu, S. Liu, J. Shi, M. Shan, H. Shi, Z. Xu, H. Deng, *Separation and Purification Technology* **2022**, 282, 120030.

[25] W. Shao, S. Liu, K. Wang, J. Niu, L. Zhu, S. Zhu, G. Ren, X. Wang, Y. Cao, H. Zhang, Y. Wang, X. Sun, F. Liu, J. He, *Separation and Purification Technology* **2024**, 333, 125872.

[26] J.-S. Wang, R.-R. Cai, S.-J. Wu, L.-Z. Zhang, *Chemical Engineering Science* **2023**, 265, 118237.
